# Quantitative Diffusion and T2 Mapping Using RF‐Modulated Phase‐Based Gradient Echo Imaging

**DOI:** 10.1002/mrm.70312

**Published:** 2026-03-11

**Authors:** Daiki Tamada, Ali Pirasteh, David F. Jarrard, Diego Hernando, Scott B. Reeder

**Affiliations:** ^1^ Department of Radiology University of Wisconsin‐Madison Madison Wisconsin USA; ^2^ Department of Medical Physics University of Wisconsin‐Madison Madison Wisconsin USA; ^3^ Department of Urology University of Wisconsin‐Madison Madison Wisconsin USA; ^4^ Carbone Cancer Center University of Wisconsin‐Madison Madison Wisconsin USA; ^5^ Department of Biomedical Engineering University of Wisconsin‐Madison Madison Wisconsin USA; ^6^ Department of Medicine University of Wisconsin‐Madison Madison Wisconsin USA; ^7^ Department of Emergency Medicine University of Wisconsin‐Madison Madison Wisconsin USA

**Keywords:** apparent diffusion coefficient, diffusion, phase, quantitative imaging biomarker, RF‐modulated gradient echo imaging

## Abstract

**Purpose:**

To introduce and evaluate the feasibility of a novel RF‐phase modulated gradient echo (GRE) method for quantitative diffusion MRI, aimed at mitigating geometric distortion and enabling high‐resolution 3D quantitative diffusion/T2 mapping as a complementary alternative to conventional DWI.

**Theory and Methods:**

The proposed phase‐based diffusion (PBD) method employs RF phase modulation to encode both diffusion and T2 information into the GRE signal phase. A closed‐form analytical model enables joint apparent diffusion coefficient (ADC) and T2 mapping via iterative reconstruction. The method's feasibility was evaluated via Bloch equation simulations, phantom experiments, and preliminary in vivo imaging studies.

**Results:**

Monte Carlo simulations revealed that PBD provides more accurate median ADC estimates at low signal‐to‐noise ratios (SNRs) compared to conventional single‐shot echo‐planar imaging (SS‐EPI), although PBD exhibited greater variability. Phantom studies demonstrated good agreement for PBD‐derived ADC values (e.g., *R* ^2^ = 0.99) with reference methods and strong correlation for PBD‐derived T2 values (e.g., *R* ^2^ = 0.89), though the latter showed some systematic bias in phantoms. In vivo results from patients with benign or malignant prostate disease demonstrated the feasibility of the PBD method to provide high‐resolution ADC and T2 maps with minimal geometric distortions relative to conventional SS‐EPI.

**Conclusion:**

PBD provides ADC and T2 maps with improved geometric fidelity in phantoms and in vivo, and offers robust median ADC estimates from noisy data based on simulations. This combination of spatial precision and noise characteristics makes PBD promising for applications such as high‐resolution DWI for prostate MRI.

## 1 Introduction

Diffusion‐weighted MRI (DWI) is an indispensable tool for numerous applications, including stroke and tumor detection, and quantitative prediction and monitoring of treatment response [1, 2, 3, 4, 5]. Current clinical DWI protocols typically rely on single‐shot echo‐planar imaging (SS‐EPI) due to its speed and robustness against physiological fluctuations. However, SS‐EPI suffers from significant geometric distortion caused by magnetic susceptibility variations, limited spatial resolution (typically 2–4 mm in‐plane), image blurring, and other challenges [6, 7, 8]. These limitations hinder the detection of small lesions and the precise delineation of tissue boundaries [9].

Multi‐shot techniques, such as readout‐segmented EPI (RS‐EPI) [10] and multiplexed sensitivity‐encoding (MUSE) [11], divide the acquisition into segments, enabling higher resolution and reduced distortion. However, these methods introduce sensitivity to inter‐shot motion, requiring complex navigator‐based correction schemes that increase scan time [8]. These methods are also limited to 2D imaging. Steady‐state methods, like diffusion‐weighted double‐echo steady‐state (DW‐DESS) [12, 13, 14, 15], offer another path to high‐resolution 3D‐DWI with minimal geometric distortion. As with all coherent steady‐state GRE approaches, they are sensitive to motion because they rely on coherent pathways and unbalanced gradient moments. While in vivo quantification has been demonstrated in specific applications (e.g., cartilage) [12, 13, 16], broader quantification remains challenging and often requires careful sequence and model tuning [17, 18, 19]. Consequently, despite the recognized clinical need for high‐resolution DWI [20, 21], lower‐resolution SS‐EPI remains the dominant clinical standard, as emerging alternative multi‐shot techniques often incur scan time penalties or require complex navigator‐based correction.

Recently, Wang et al. investigated T2 mapping by encoding T2 information into the signal phase using RF‐modulated gradient echo (GRE) MRI [22]. Their work highlighted the potential of phase for quantitative relaxometry. Inspired by this, we have demonstrated initial feasibility of phase‐based diffusion (PBD) [23, 24]. PBD uses small RF phase increments and variable spoiler gradient moments to encode ADC and T2 predominantly in the GRE signal phase, a distinct encoding mechanism from conventional DWI or DW‐DESS. In this work, we derive a closed‐form steady‐state model with a diffusion term that enables joint ADC and T2 estimation from images acquired with small RF phase increments. Furthermore, we have implemented this approach with radial stack‐of‐stars (SoS) trajectories to improve motion robustness for PBD [24]. Therefore, PBD may be a viable alternative, similar to DW‐DESS as a coherent steady‐state method, and thus motion‐sensitive, but different in its signal encoding and modeling strategy.

This study introduces and evaluates the feasibility of PBD, combined with a SoS radial trajectory. This novel approach provides quantification of both ADC and T2 of tissue with improved spatial resolution, minimal distortion, and reduced motion‐related challenges associated with conventional DWI methods. We also derive a novel closed‐form analytical expression for the RF‐modulated GRE signal with diffusion, providing a theoretical foundation for this technique.

## 2 Theory

### 2.1 Signal Representation

In this study, PBD is achieved using RF phase‐modulated gradient‐echo acquisitions with varying gradient crusher moments that encode diffusion information into the signal phase. We recently showed that both diffusion and T2 weighting can be encoded into the complex MR signal using GRE with quadratically incremented RF phase [23]. Small RF phase increments (e.g., 1°–3°) preserve transverse magnetization and encode diffusion and T2 weighting into the signal phase. The steady state is a coherent sum of many echo pathways created by successive excitations. A small RF phase increment provides a progressive phase increase on later pathways, which experience longer effective echo times accumulate larger relative phase. Diffusion and T2 cause greater attenuation of the longer‐lived pathways compared to the shorter‐lived ones. The net complex sum therefore rotates in the complex plane, so the phase increases monotonically with both T2 and ADC. To characterize the signal, we derive a novel closed‐form equation for the steady‐state of PBD signals based on the approaches proposed by Sobol and Hennig [25, 26]. We consider a sequence with RF pulses with flip angle (*α*), RF phase (*ϕ*), and repetition time (TR). RF phase modulation is performed by incrementing the transmit RF phase ϕ quadratically such that ϕ(n)=ϕ(n−1)+nθ, where *θ* is the phase increment of the *n* ^th^ RF excitation. Rectangular crusher gradients with a gradient moment of G provide diffusion weighting. The steady‐state complex signal (*S*) after RF excitation can be expressed as

(1) $$S=\beta \left[\eta X\left(-1\right)+i\left(\eta^{2}-\epsilon \left(X\left(-1\right)-\epsilon \right)\right)\right]$$

with

(2) $$\begin{array}{l} \beta =\frac{\left(1-Y\left(0\right)\right)M_{0}\text{sinα}}{\begin{array}{c} \left(X\left(-1\right)-\epsilon \right)\left[X\left(-1\right)\left(\text{cosα}-Y\left(0\right)\right)+\epsilon \left(1-Y\left(0\right)\text{cosα}\right)\right]-\eta^{2}\left(1-Y\left(0\right)\text{cosα}\right) \end{array}} \end{array}$$

where $M_{0}$ is the proton density, and ϵ and η are real coefficients that are defined as

(3) $$\epsilon +i\eta =-\frac{\Omega_{22}}{\Omega_{21}}$$

where ϵ and η can be determined from the matrix elements, $\Omega_{21}$ and $\Omega_{22}$, of the recursive matrix equation:

(4) $$\left[\begin{array}{ll} \Omega_{11} & \Omega_{12}\\ \Omega_{21} & \Omega_{22} \end{array}\right]=\Psi_{L}\Psi_{L-1}\ldots \Psi_{1},$$

with the matrix $\Psi_{l}$ defined as

(5) $$\begin{array}{ll} \Psi_{l} & =\frac{1}{X\left(-l-1\right)\cdot \left(-1+Y\left(l\right)e^{2\mathrm{iCl}}\right)}\cdot \left[\begin{array}{cc} X\left(l-1\right)\cdot X\left(-l-1\right)\cdot \left(Y\left(l\right)e^{2\mathrm{iCl}}-\cos\alpha \right)\cdot \sec^{2}\frac{\alpha }{2} & -X\left(-l-1\right)\cdot \left(1+Y\left(l\right)e^{2\mathrm{iCl}}\right)\cdot e^{-2\mathrm{iC}l^{2}}\cdot \tan^{2}\frac{\alpha }{2}\\ X\left(l-1\right)\cdot \left(1+Y\left(l\right)e^{2\mathrm{iCl}}\right)\cdot e^{2\mathrm{iC}l^{2}}\cdot \tan^{2}\frac{\alpha }{2} & \left(-1+Y\left(l\right)e^{2\mathrm{iCl}}\cos\alpha \right)\cdot \sec^{2}\frac{\alpha }{2} \end{array}\right], \end{array}$$

where *C* =θ/2, and *l* = 1, …, *L*, with *L* chosen sufficiently large such that the signal contribution from configurations beyond *L* is negligible due to attenuation from diffusion and T2 decay. In this study, we use the configuration sum at *L* = 8. Limiting the configuration sum is a modeling choice for fidelity/efficiency and does not affect the method's inherent motion sensitivity. *X* and *Y* are T2 and T1 relaxation factors, and include the diffusion term defined as

(6) $$\begin{array}{cc} X\left(l\right) & =e^{-\frac{t}{T2}}\cdot e^{-D\gamma^{2}G^{2}t^{3}\left(l^{2}+l+\frac{1}{3}\right)}\\ Y\left(l\right) & =e^{-\frac{t}{T1}}\cdot e^{-D\gamma^{2}G^{2}t^{3}l^{2}} \end{array}$$

where *t* is the gradient pulse duration, *D* is the diffusion coefficient, γ is the gyromagnetic ratio, *l* is the configuration index described by Sobol and Gauntt [25] Equations (1) and (2) describe how diffusion is encoded into GRE signal phase in addition to T2. An increased crusher gradient moment in the presence of diffusion introduces intravoxel phase dispersion that attenuates the coherent transverse sum, which is accounted for by the diffusion term. The complete derivation of Equations ((1), (2), (3), (4), (5), (6)) is included in Supporting Information S1. A MATLAB implementation of these equations is also available (Data Availability Statement). Also, validation of the equation with Bloch equation simulation is included in Supporting Information S2.

### 2.2 Reconstruction of ADC and T2 Maps

Quantitative ADC and T2 maps from the acquired PBD signals are reconstructed using an iterative optimization approach with Total Variation (TV) and Tikhonov (L2) regularization. This approach stabilizes the process for physical solutions: TV regularization reduces noise while preserving boundaries [27], and L2 regularization further stabilizes the solution, ensuring physical plausibility [28], particularly by mitigating noise amplification. The validity of the regularization parameters and their effects on image quality are summarized in Supporting Information S3.

Our approach begins by extracting phase information from four passes acquired with different gradient moments and RF phase increments. The complex MR images acquired with low ($L_{+}$) and high ($H_{+}$) gradient moments using an RF phase increment +*θ* can be expressed as:

(7) $$H_{+}\left(r\right)=He^{i\left({∆}_{H}+\varphi_{H}\right)},$$

(8) $$L_{+}\left(r\right)=Le^{i\left({∆}_{L}+\varphi_{L}\right)},$$

where ${∆}_{H}$ and ${∆}_{L}$ denote background phase errors due to B0 inhomogeneity, susceptibility effects and eddy currents caused by crusher gradients with low and high gradient moments, respectively. The terms $\varphi_{L}$ and $\varphi_{H}$ represent the phase components containing the desired diffusion and T2 information.

In order to eliminate the background phase errors, we acquire two additional images with identical gradient moments but with opposite polarity of RF phase increments (−*θ*). These additional acquisitions can be expressed as:

(9) $$H_{-}\left(r\right)=He^{i\left({∆}_{H}-\varphi_{H}\right)}$$

(10) $$L_{-}\left(r\right)=Le^{i\left({∆}_{L}-\varphi_{L}\right)}$$

From Equations ((7), (8), (9), (10)), the phase values without background phase errors can be derived as follows:

(11) $$\varphi_{H}\left(r\right)=\frac{∠\left(H_{+}\cdot H_{-}^{*}\right)}{2}$$

(12) $$\varphi_{L}\left(r\right)=\frac{∠\left(L_{+}\cdot L_{-}^{*}\right)}{2}$$

Using the set of extracted phases ($\varphi_{H}$ and $\varphi_{L}$), ADC and T2 values are estimated using the algorithm described as follows. At each gradient moment level, we acquire opposite RF phase increments (±*θ*) and form phase differences $\varphi_{L}$ and $\varphi_{H}$, which remove background phase. For our operating range, the model‐predicted $\varphi_{L}$ and $\varphi_{H}$ values remain between 0° and 90°, and for this reason we do not observe spatially varying phase wraps.

The reconstruction algorithm formulates the parameter estimation as an optimization problem that minimizes a composite cost function. This function consists of data fidelity terms measuring the discrepancy between measured and model‐predicted phases, spatial TV regularization terms enforcing smoothness in parameter maps, and additional L2 constraints ensuring physically meaningful solutions. Mathematically, we seek to minimize:

(13) $$\begin{array}{ll} J\left(x_{1},x_{2}\right) & ={\left\|f_{H}\left(x_{1},x_{2}\right)-\varphi_{H})\right\|}^{2}+{\left\|f_{L}\left(x_{1},x_{2}\right)-\varphi_{L})\right\|}^{2}\\ & \;+\lambda \cdot \left(\mathrm{TV}\left(x_{1}\right)+\mathrm{TV}\left(x_{2}\right)\right)+\beta \cdot \left({\left\|x_{1}\right\|}^{2}+{\left\|x_{2}\right\|}^{2}\right), \end{array}$$

where $x_{1}$ and $x_{2}$ represent ADC and T2 maps respectively, $f_{H}$ and $f_{L}$ are the model predictions for high and low gradient moments using Equation (1), TV denotes the total variation operator, and λ and β are regularization parameters for TV and L2 terms.

The minimization problem (Equation 13) was solved using an iterative, gradient‐based optimization algorithm. To accelerate convergence, adaptive step sizes were determined using the Barzilai–Borwein method [29]. At each iteration, the gradients of the data fidelity terms were efficiently computed using a pre‐calculated cubic spline interpolant. This interpolant, generated using MATLAB's csapi based on Equation (1), encodes the relationship between ADC/T2 values and expected signal phases, allowing for rapid evaluation of the model prediction and its gradient. The iteration continues until convergence, assessed by relative changes in the objective function. We used very small regularization coefficients ($\lambda =\beta =1.0\times 10^{-6}$) so the terms do not confound quantification. A detailed pseudo‐code of this algorithm is provided in the Supporting Information S4. In addition, a MATLAB implementation is available (see Data Availability Statement).

### 2.3 Determination of *b* ‐Value

In PBD, the *b*‐value is not well‐defined because the signal consists of multiple echoes, which experience different effective echo times and diffusion times. Bieri et al. determined an approximate “effective *b*‐value” for DW‐DESS to compare DW‐DESS with established methods. They calculated it by forcing the signal attenuation to fit the Stejskal–Tanner equation [13]. Similarly, we defined *b*‐value of PBD signals by fitting simulated signals to the Stejskal–Tanner equation [30]. As explained in the previous study [23] and Supporting Information S5, diffusion‐ and T2‐weighted information is encoded primarily into the real components of the signals [24, 31]. Therefore, the estimated *b*‐value ($\hat{b}$) can be calculated by solving the following problem:

(14) $$\hat{b}=\underset{b,S_{0}}{\text{argmin}}\left\|\sum_{D=0}^{2000}R\left[\hat{S}\left(D\right)\right]-S_{0}e^{-\mathrm{bD}}\right\|$$

where $\hat{S}$ is the calculated PBD signals using Equation (1), and $S_{0}$ and *b* are the magnitude and *b*‐value of the Stejskal–Tanner equation. Since the real component of PBD signal depends on both diffusion and T2 (Equation 6), estimating an effective *b*‐value ($\hat{b}$) using the Stejskal–Tanner model requires the assumption of a representative T2 value. For this estimation, we used a fixed T2 of 120 ms, chosen as a reasonable approximation for brain (T2˜80–110 ms [32]) and prostate (T2˜80–210 ms [33, 34]). While this is not strictly equivalent to a conventional DWI *b*‐value, it is intended to reflect a comparable level of diffusion weighting. Fitting examples for the effective *b*‐value are shown in Supporting Information S6.

## 3 Methods

### 3.1 Acquisition Strategy and Reconstruction

**FIGURE 1:**
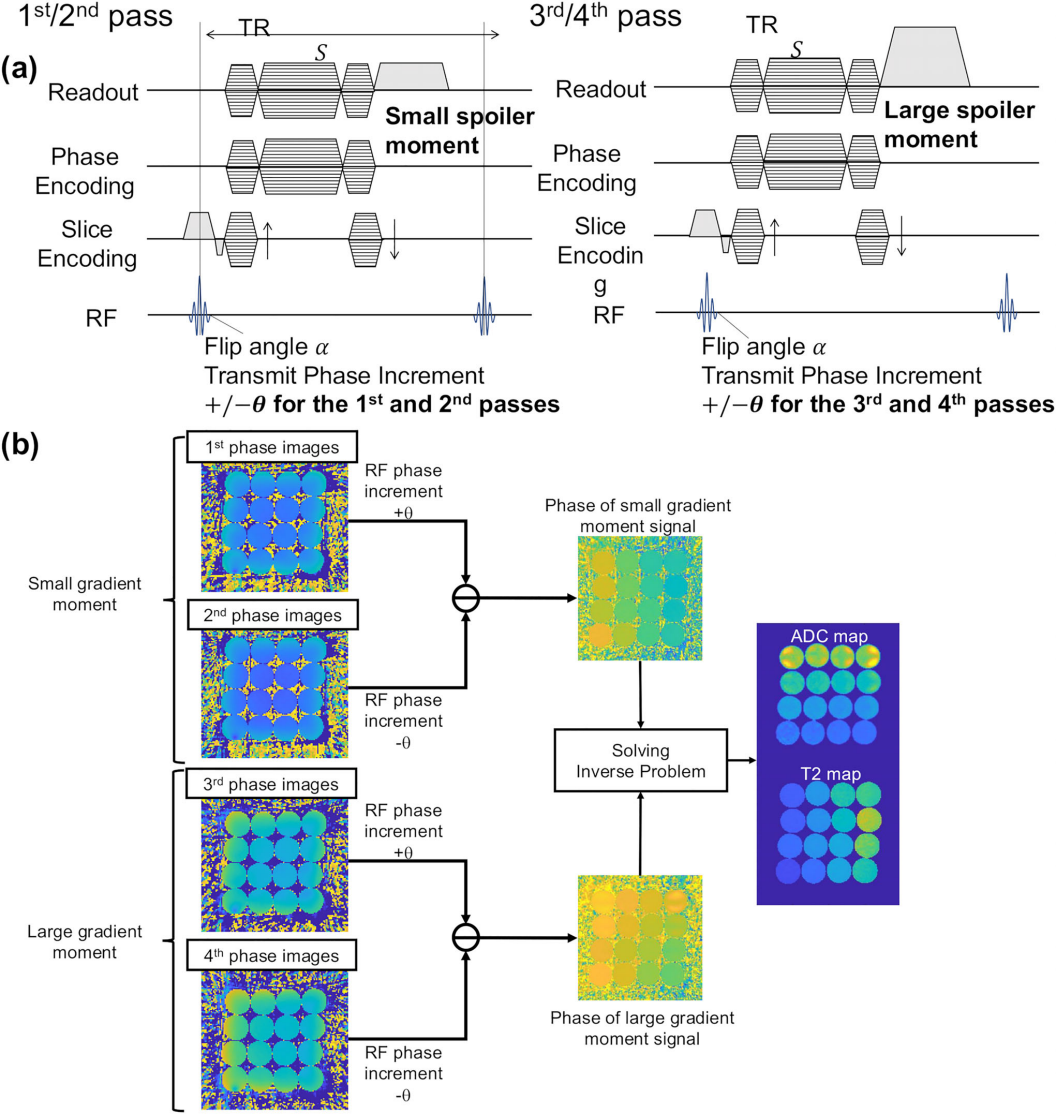
Schematic diagrams of the proposed Phase‐Based Diffusion (PBD) method. (a) Pulse sequence diagram illustrating the RF‐phase modulated GRE sequence with alternating gradient moments (*G*_L_/*G*_H_) and RF phase increments (+θ/−θ), implemented with a stack‐of‐stars trajectory for motion robustness. (b) Reconstruction workflow diagram showing the iterative optimization process used to estimate T2 and ADC maps from the acquired phase difference signals (*φ*_L_, *φ*_H_), incorporating prior information and spatial regularization (TV, L2).

Image acquisition was performed using an RF phase‐modulated GRE sequence with a 3D radial SoS trajectory [35] to reduce motion sensitivity, as shown in Figure 1a. For phantom experiments, a Hamming‐filtered sinc RF pulse was used, while binomial water‐excitation pulses were used for in vivo studies to suppress fat signals. Binomial pulses have been widely adopted in steady‐state imaging to provide robust fat suppression and prevent streaking artifacts while preserving a stable steady state. To maintain the steady state of the transverse magnetization, readout gradients were rewound along all axes after each spoke acquisition, ensuring consistent gradient spoiling conditions along physical gradient axes before each RF pulse. As detailed in the Theory section, a four‐pass acquisition scheme was performed (Figure 1b). This involved two passes with a low gradient moment and two passes with a high gradient moment to provide differential diffusion weighting. Within each gradient moment setting, two acquisitions were performed using opposite RF phase increments (±2°). Finally, ADC and T2 maps were calculated from the phase components using the iterative regularized algorithm described in Theory. No explicit T2* term was included in the reconstruction model. The two outermost slices at each end of the volume were discarded to mitigate any edge‐related artifacts caused by imperfect slab excitation profiles.

### 3.2 Signal Behavior Simulations

To characterize the signal behavior of the PBD sequence, a series of numerical simulations were performed using the derived closed‐form solution. The signal phase with high gradient moment (16.3 s mT/m) was calculated across a wide range of physiologically relevant tissue properties, including ADC (0–2000 μm^2^/s), T2 (50–300 ms), T1 (700–1300 ms), as well as sequence parameters, including flip angle (0°–40°), RF phase increments (1°, 2°, 4°). Additionally, phase responses at both low (2.33 s mT/m) and high (16.3 s mT/m) gradient moments were calculated across a grid of ADC and T2 values for a fixed flip angle of 20° and an RF phase increment of 2°.

To quantify a potential bias introduced by B1+ inhomogeneity, we simulated PBD signals using a range of flip angles to model B1+ variations. These signals, generated using the standard low (2.33 s mT/m) and high (16.3 s mT/m) gradient moments, were then processed with our reconstruction algorithm without regularization to estimate the resulting ADC and T2 values.

To explain the PBD encoding mechanism, the steady‐state signals at low (L) and high (H) gradient moments (2.33, 16.3 s mT/m) were simulated across a grid of ADC (0–2000 μm^2^/s) and T2 (50–300 ms) values. Using a fixed TR (10 ms), flip angle (20°) and RF phase increment (2°), the phase difference (∠L−∠H), magnitude ratio (‖H‖/‖L‖), real (R(H)/R(L)) and imaginary (I(H)/I(L)) component ratios between the low and high gradient moment states were calculated.

Unless otherwise specified, the simulations were performed using the following baseline parameters/settings: TR = 10 ms, flip angle (20°), RF phase increment = 2°, T1 = 1000 ms, T2 = 100 ms.

### 3.3 Noise Performance Simulation

Monte Carlo simulations were performed to compare the quantitative accuracy and noise characteristics of the PBD against reference methods (SS‐EPI and single‐echo spin‐echo [SESE]), particularly at low SNR conditions. For SS‐EPI, we used one (EPI 1‐dir) and three (EPI 3‐dir) diffusion‐encoding directions. The simulations used a single‐voxel model with three sets of ground truth ADC (800, 1300, 1800 μmm^2^/s) and T2 (100, 120, 200 ms) values representative of prostate tissues, roughly corresponding to cancer, normal central gland, and normal peripheral zone tissues at 3 T [34, 36, 37, 38].

Noiseless signals were generated for each method using their respective physical models, based on the sequence parameters from the in vivo volunteer experiments (Table 1). To ensure a fair and clinically relevant comparison, the subsequent noise analysis was performed under a matched voxel size and normalized to a fixed total acquisition time (10 min). This normalization involved three steps: First, to avoid confounding by arbitrary signal scaling, the baseline signal amplitudes were determined directly from the physical models for each sequence. Second, the effective number of signal averages (NSA) achievable for each method within the total time was calculated. Third, complex Gaussian noise was added, with the noise variance scaled to account for differences in receiver bandwidth (BW), the calculated NSA, and the number of slice‐encoding ($N_{s}$). Specifically, noise variance was scaled to be proportional to BW, 1/NSA, and 1/$N_{s}$. Simulations were performed across a range of effective SNRs from 3 to 50 [39].

**TABLE 1:** Sequence parameters.

	Phantom			Patient		
Parameters	PROPELLER DWI	MESE	PBD	SS‐EPI DWI	FSE T2W	PBD
TR (ms)	2000	1200	10.9	4500	3738	10.9
(Effective) TE(s) (ms)	50	8.5–68	1.5	60	106	2.9
Flip angle	90°	90°	20°	90	111	20°
FOV	24 × 24 cm^2^	18 × 18 × 12.8 cm^3^	24 × 24 × 9.6 cm^3^	18 × 18 cm^2^	26 × 26 cm^2^	18 × 18 × 12.8 cm^3^
Matrix size	160 × 160	256 × 128	160 × 160 × 32	120 × 120	384 × 256	160 × 160 × 32
Number of slices	28	NA	NA	22	43	NA
Slice thickness (mm)	3	4	4	4	2.4	4
Bandwidth (Hz/px)	488	488	488	1953	244	325
(Effective) *b*‐value (s/mm^2^)	1000	NA	24/668	1500	NA	25/675
Gradient moment (s mT/m)	NA	NA	2.33/9.33	NA	NA	2.38/16.3
RF phase increment	NA	NA	+2°/−2°	NA	NA	+2°/−2°
Acquisition time (min)	1:28	3:25	4:25	4:48	2:37	4:45

For each condition, 100 000 noisy signals were generated. The resulting parameter estimates were calculated using the appropriate algorithm for each method. For SS‐EPI, ADC was estimated from the magnitude using the standard log‐ratio: ADC = $-\frac{1}{b_{H}}\cdot \log\frac{S_{b_{H}}}{S_{b0}}$, where $S_{b0}$ and $S_{b_{H}}$ are magnitudes at b = 0 and $b_{H}$, respectively. For SESE, T2 was estimated by fitting S(TE) = S0·exp(−TE/T2) to the multi‐echo magnitudes using nonlinear least squares. The proposed ADC/T2 estimation algorithm without regularization terms was used for PBD to assess single‐voxel performance. The mean, median, and standard deviation of the resulting ADC/T2 distributions were calculated and plotted against the time‐normalized SNR of the reference SS‐EPI or SESE. A detailed implementation of this time‐normalized simulation is available in the provided code repository.

### 3.4 CRLB Analysis

A Cramér‐Rao Lower Bound (CRLB) analysis [40] was performed to identify the factors limiting parameter precision. We modeled the measurement vector $y=\left[\varphi_{L},\varphi_{H}\right]$ and computed the Jacobian $J=\frac{\partial y}{\partial \left[D,T2\right]}$ by central differences of the analytical model at low and high gradient moments. The Fisher information is $F=J^{T}\Sigma^{-1}J$ and $\text{CRLB}=\mathrm{diag}\left(F^{-1}\right)$ with precision reported as standard deviations SD=√CRLB. Two noise models were used. (i) Amplitude‐weighted, reflecting a realistic scenario where phase variance is inversely proportional to the signal magnitude: one complex noise level is chosen so that the $L_{+}$/$L_{-}$ signal has $\mathrm{SN}R_{\mathrm{ref}}$ at each design point. (ii) Equal‐variance: both final phase measurements have $\mathrm{Var}\left(\varphi_{L}\right)=\mathrm{Var}\left(\varphi_{H}\right)\approx 1/\left(2\cdot \mathrm{SN}R_{\mathrm{ref}}^{2}\right)$. We report the standard‐deviation amplification ratio $r=SD_{\mathrm{amp}}/SD_{\mathrm{eq}}$ to attribute limits to amplitude (*r* > 1) or conditioning (*r*≈1). Unless noted: TR = 10.9 ms, *θ* = 2°, T2 = 120 ms, ADC = 1300 μm^2^/s, $\mathrm{SN}R_{\mathrm{ref}}$ = 10.

### 3.5 Phantom Construction

A set of phantoms was constructed to validate the proposed diffusion quantification method. Specifically, a total of 16 vials were constructed with four rows of increasing polyvinylpyrrolidone (PVP) [41] to encode diffusion coefficients. Increasing values of PVP including 10%, 20%, 30%, and 40% w/w were added to each set of vials. For each of the four rows, an increasing concentration of MnCl_2_ was added to modulate T2. Values of MnCl_2_ ranging from 0.01 to 0.08 mM were added to obtain a range of T2 values similar to that typically seen in vivo [42].

### 3.6 Phantom Experiments

All phantom studies were performed on a 3.0 T clinical MRI system (SIGNA Premier, GE Healthcare, Waukesha, WI) using a 48ch head coil (AirCoil, GE Healthcare, Waukesha, WI). First, reference values for ADC and T2 were obtained using conventional sequences. ADC maps of the phantom were acquired using a 2D PROPELLER fast spin echo (FSE) sequence [43] to minimize susceptibility artifacts at phantom‐air interfaces that commonly degrade conventional EPI‐based measurements. T2 mapping was performed using a two‐dimensional (2D) multi‐echo spin‐echo (MESE) sequence. Parameters from conventional acquisitions were estimated in MATLAB (R2022b; MathWorks, Natick, MA) by fitting signal intensities to mono‐exponential decay models on a pixel‐by‐pixel basis. ADC and T2 maps were reconstructed using the proposed iterative algorithm.

ADC and T2 maps of the same phantom were acquired using the proposed PBD sequence. The acquired k‐space data were reconstructed using the CG‐SENSE [44] algorithm implemented with BART toolbox [45]. Acquisition parameters for ADC and T2 mapping are found in Table 1, and Supporting Information S8 and S9.

To evaluate geometric distortion under strong susceptibility, we constructed a phantom containing background tissue (2% w/w agar, 10% w/w PVP) and two cylinders: one mimicking the prostate (1% w/w agar, 40% w/w PVP) and one air‐filled to simulate the rectum. PBD, SS‐EPI, and reference T2W FSE were acquired using clinical parameters (see Section 3.7). Geometric fidelity was quantified by comparing the prostate‐mimicking cylinder diameter to the T2W reference.

### 3.7 In Vivo Experiments

Informed consent was obtained prior to imaging. In vivo imaging was performed on 3.0 T clinical MRI systems (SIGNA Premier/Discovery MR750, GE Healthcare, Waukesha, WI) using a phased array torso coil (AirCoil, GE Healthcare, Waukesha, WI). All subjects were recruited from an Institutional Review Board (IRB)‐approved database, and informed consent was obtained prior to imaging.

To evaluate geometric fidelity in a clinical setting, a patient with benign prostatic hyperplasia (BPH) was imaged. This subject was selected to assess performance in the presence of typical pelvic susceptibility interfaces (e.g., rectal gas). Diffusion‐weighted images were acquired and reconstructed using PBD and SS‐EPI and compared visually against T2W FSE images to assess for anatomical distortion.

To demonstrate the potential clinical application, two patients with known prostate cancer were also prospectively recruited. The PBD sequence was acquired following completion of the clinical imaging protocol for each patient. For the patient protocol, reduced‐FOV SS‐EPI (FOCUS, GE Healthcare) with *b* = 0 and *b* = 1500 s/mm^2^ and three orthogonal MPG directions was used. This is the standard DWI sequence for the prostate at our institution. Because the combination of high b value and reduced FOV lowers SNR [46], the protocol includes multiple averages at low and high b values. Consequently, total DW‐EPI time in patients is longer than in the volunteer.

ADC and T2 maps were reconstructed as described previously. Additionally, synthetic diffusion‐weighted and T2‐weighted images were generated based on the estimated ADC/T2 values. Diffusion‐weighted images with a *b*‐value of 1500 s/mm^2^ were calculated using a synthetic diffusion imaging approach [47] by multiplying T2‐weighted images with a mono‐exponential signal decay model. T2‐weighted images were generated based on the phase‐based method [31].

Image contrast and ADC maps obtained from PBD were visually compared with those from the conventional clinical sequences acquired during the same session, specifically standard SS‐EPI and T2‐weighted FSE (T2W‐FSE). Detailed acquisition parameters for the PBD sequence and the relevant clinical sequences are provided in Table 1.

To perform a quantitative comparison of the PBD and reference methods in vivo, region of interest (ROI) analyses were conducted on the acquired quantitative maps. ROIs were placed at suspected lesions, as well as normal‐appearing tissue in the peripheral (PZ) and transition zones (TZ). For each ROI, the mean, median, and standard deviation of the pixel values were calculated for both the PBD and the corresponding reference sequences.

## 4 Results

### 4.1 Signal Behavior Simulations

**FIGURE 2:**
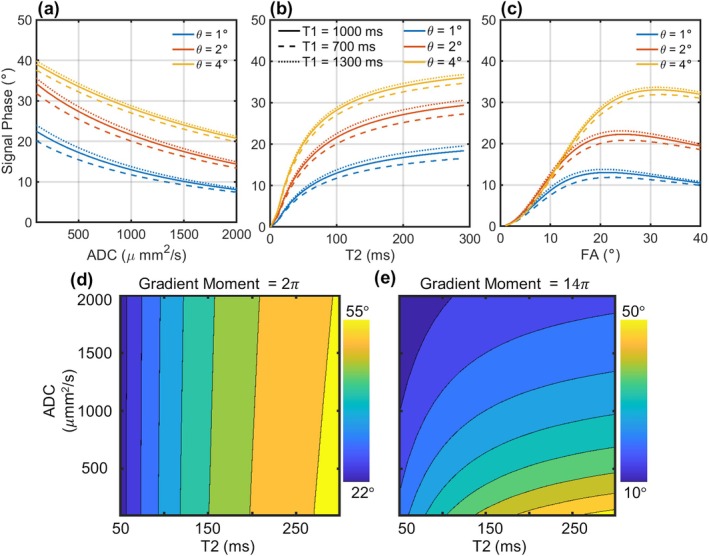
Simulated signal phase dependency on (a) T2, (b) ADC, and (c) flip angle (FA) for varying small RF phase increments (1°, 2°, 4°). Simulations were performed using high (14π) gradient moments with T1 of 700 (dashed line), 1000 (solid line), 1300 ms (dotted line). A fixed TR of 10 ms and TE of 0 ms were used. A monotonic increase in phase with T2 and ADC (at high gradient moment) is observed for RF phase increments between 1° and 4°. Also, phase response is relatively insensitive to T1. Signal phase response as a function of T2 and ADC with (d) low and (e) high gradient moment and fixed FA (20°) and RF phase increments (2°) demonstrated that ADC encodes a wide range of T2 into signal phase.

Simulations demonstrate that the PBD signal phase has distinct dependencies on tissue properties and sequence parameters (Figure 2a–c). T1 was confirmed to have a minimal effect on the signal phase. As shown in Figure 2d,e, the signal phase varies significantly with T2 at both low and high gradient moments. In contrast, ADC has a strong influence on the signal phase primarily when the larger gradient moment is applied.

**FIGURE 3:**
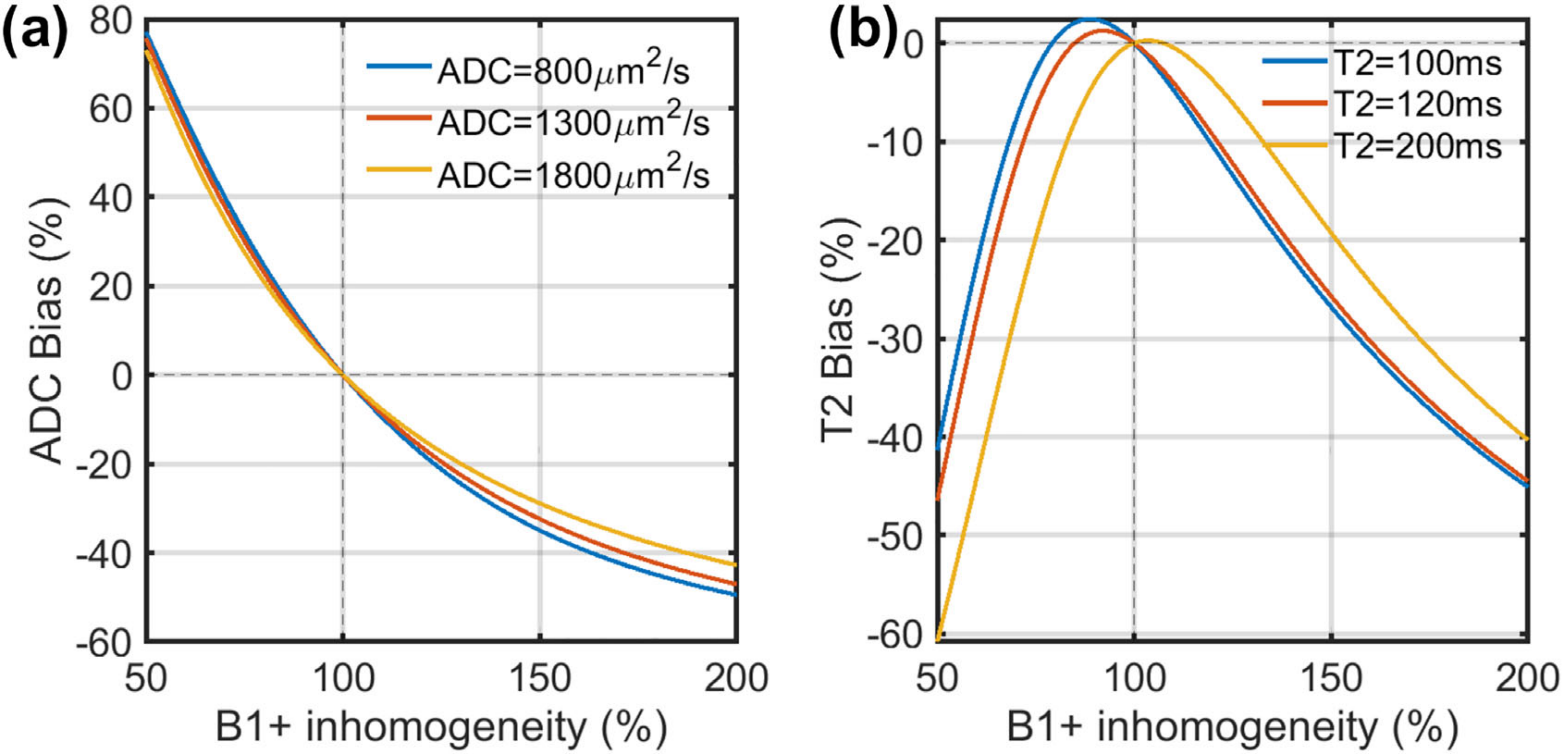
Simulated bias in ADC and T2 estimation resulting from B1+ inhomogeneity. (a) Percentage bias in the estimated Apparent Diffusion Coefficient (ADC) as a function of B1+ inhomogeneity. The ADC is overestimated when B1+ is below 100% and underestimated when it is above 100%. (b) The T2 bias is nonlinear and shows significant underestimation at both low (< 75%) and high (> 125%) B1+ levels.

Figure 3 presents simulated percentage bias for ADC and T2 estimates due to B1+ inhomogeneity. ADC values are overestimated when B1+ is below 100% and underestimated when it exceeds 100%. Bias in T2 estimation also exhibits a nonlinear relationship with B1+ inhomogeneity, demonstrating underestimation at both lower and higher B1+ levels.

**FIGURE 4:**
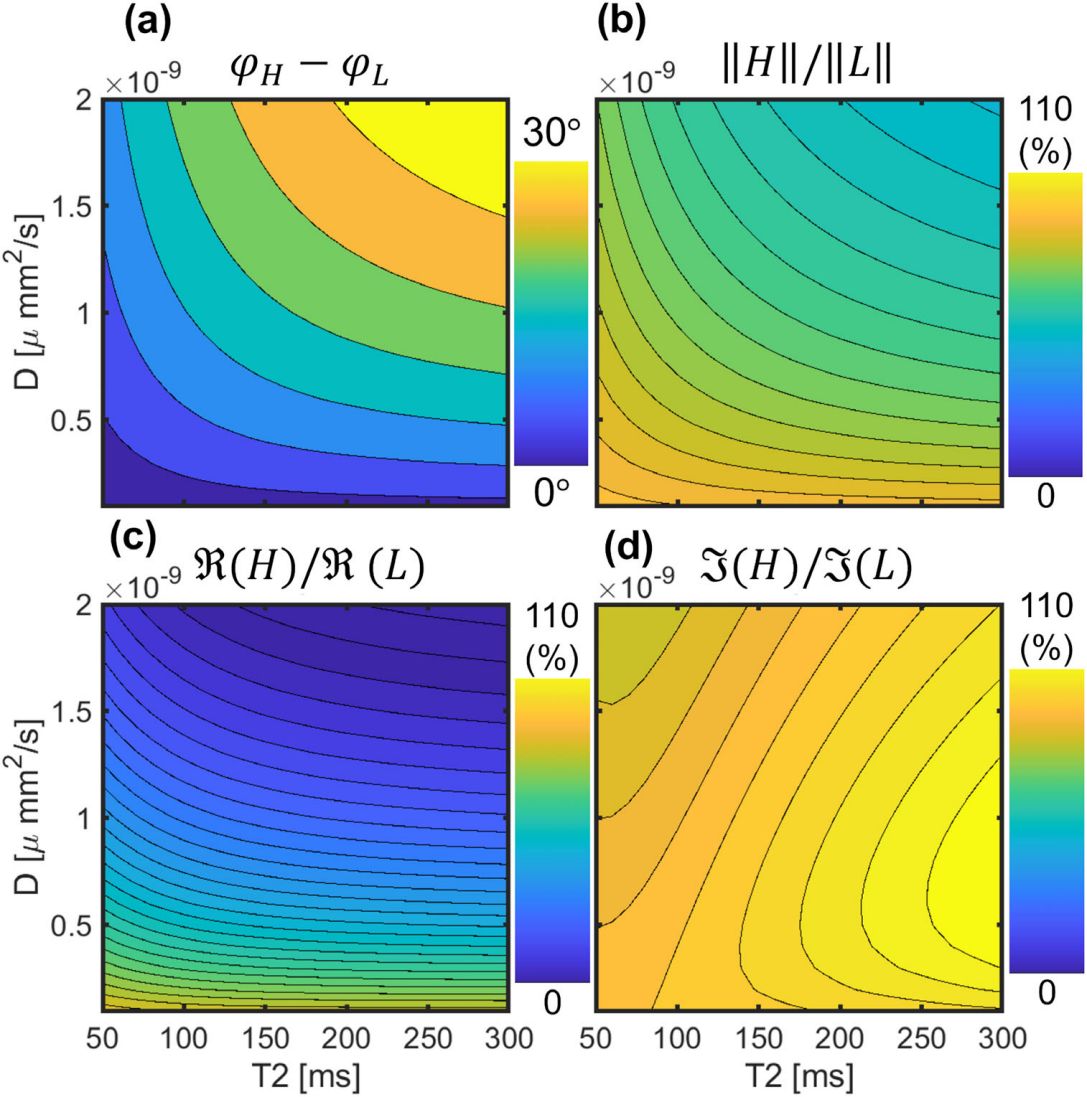
Mechanism of phase‐based encoding revealed by complex signal analysis. Simulations show the change between high and low gradient moment signals across a range of ADC and T2 values. The real component ratio (c) varies strongly with both parameters, while the imaginary component ratio (d) is much less sensitive. Consequently, the magnitude ratio (b) follows the behavior of the real component, and the phase difference (a) increases monotonically with both ADC and T2.

Analysis of the complex signal components reveals the mechanism of this phase‐based encoding (Figure 4). The simulations demonstrate that sensitivity to ADC and T2 is predominantly carried by the signal's real component. The resulting attenuation of the real component with increasing ADC and T2, while the imaginary component remains relatively stable, leads to the monotonic increase observed in the overall signal phase.

### 4.2 Noise Simulation Results

**FIGURE 5:**
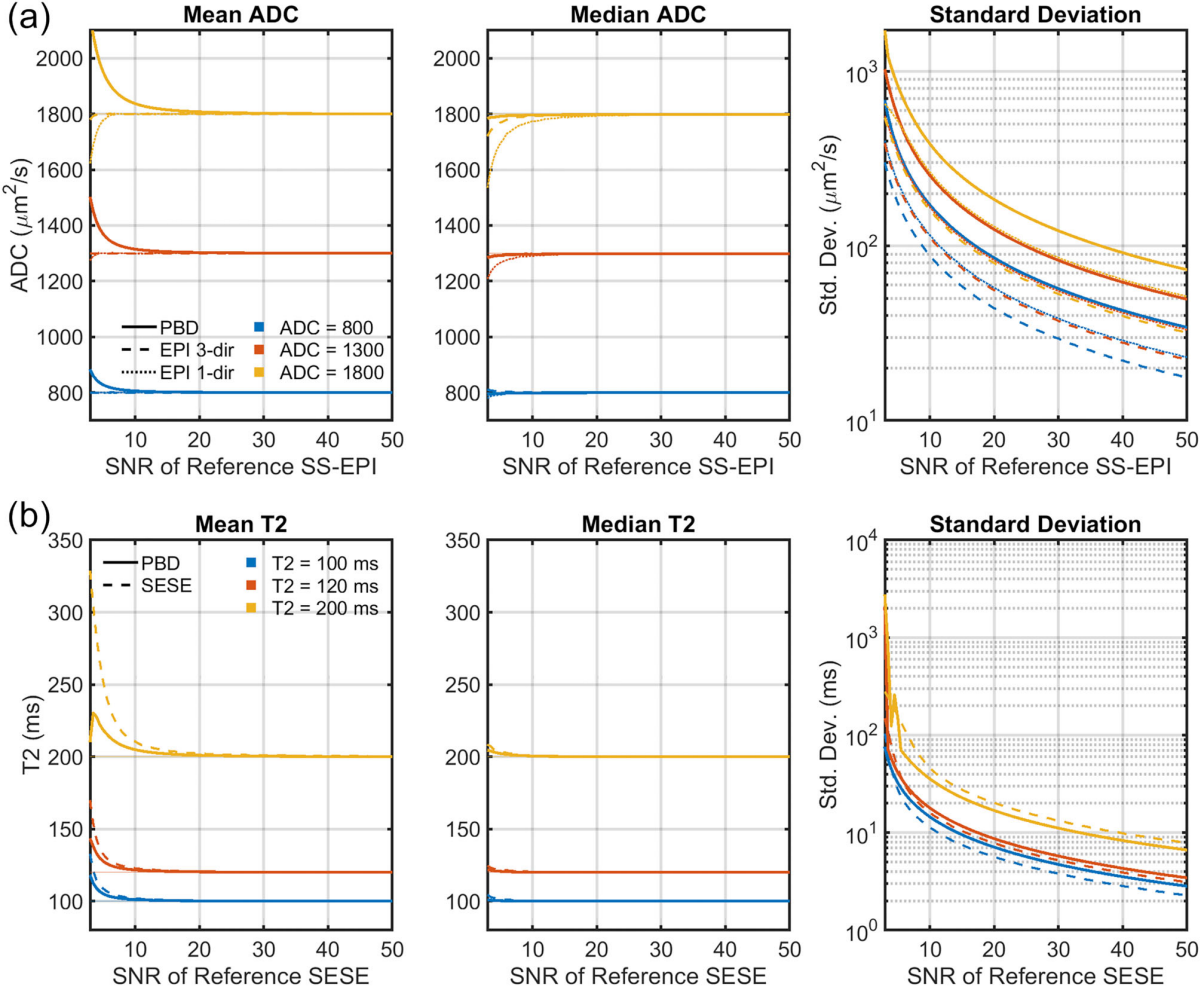
Time‐normalized Monte Carlo simulation results comparing the noise performance of the proposed PBD method (solid lines) and reference methods (dashed lines) for (a) ADC (b) T2 estimation. (a) The mean relative bias demonstrates overestimation for PBD and underestimation for SS‐EPI at low SNR. On the other hand, the median PBD estimate remains robust and nearly unbiased, in contrast to the SS‐EPI median, which is biased by the Rician noise floor. Precision is lower for PBD than for the time‐matched SS‐EPI. (b) T2 estimation of the PBD compared to SESE. The PBD median T2 estimate shows lower bias than the reference method and comparable variability.

Figure 5 shows the quantitative accuracy and precision of the proposed PBD method compared to reference methods, as evaluated using time‐normalized Monte Carlo simulations across a range of SNRs, ADC, and T2 values. Mean ADC estimates showed bias for both methods at very low SNR (SNR < 10) (Figure 5a). The EPI 1‐dir mean was substantially underestimated, consistent with the Rician floor effects, while the EPI 3‐dir mean, benefiting from the noise reduction of averaging, exhibited minimal bias. The PBD method, while avoiding the Rician floor effects, exhibited overestimation bias in the mean ADC at very low SNR, likely attributable to the non‐linear relationship between phase and ADC combined with noise. In contrast, the median estimated ADC highlighted a key difference in quantitative accuracy between the methods. The median ADC derived from PBD remained close to the true ADC value across all tested SNRs and truth ADC values, indicating minimal bias in the typical estimate. Conversely, the median ADC from SS‐EPI showed underestimation bias that worsened considerably at lower SNRs, mirroring the trend observed in the mean but confirming that the central tendency of the SS‐EPI estimates is severely affected by noise bias. The standard deviation of the PBD‐derived ADC estimates was consistently higher than that of SS‐EPI across the simulated SNR range: by a factor of 1.5–14.0 compared to EPI 1‐dir, and by a factor of 1.9–15.5 compared to EPI 3‐dir. In summary, while PBD provides a more accurate median estimate at low SNR, the per‐voxel variability is higher than SS‐EPI under the same total acquisition time. The analysis of T2 estimation revealed different trends compared to the ADC results (Figure 5b). Both the mean and median T2 from PBD showed lower bias than the SESE reference at low SNR. The variability of PBD was comparable to that of SESE at moderate‐to‐high SNR. However, while the mean and median estimates remained stable, the standard deviation increased sharply at SNRs below 5, indicating a significant loss of precision in this noise‐limited regime.

### 4.3 CRLB Analysis

**FIGURE 6:**
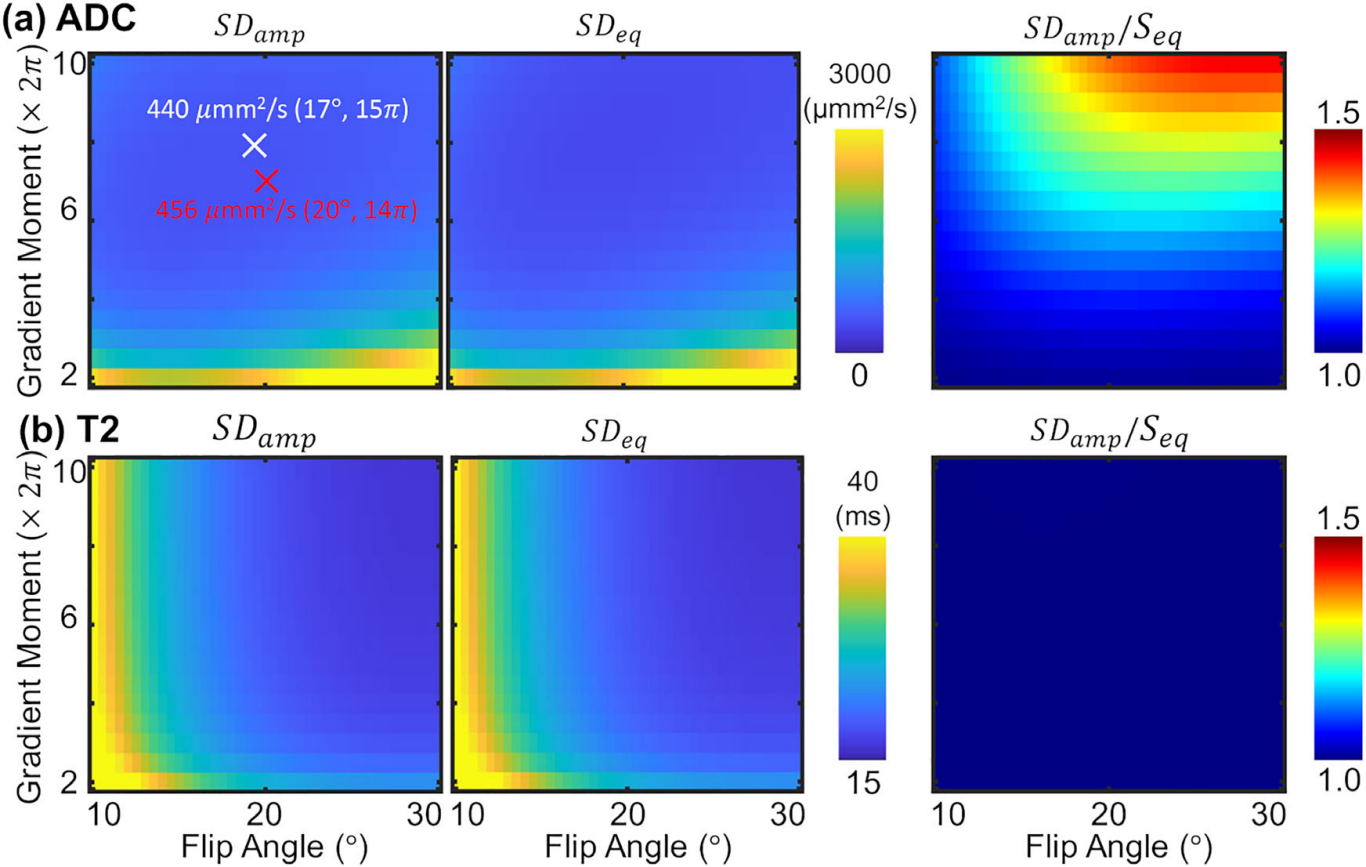
CRLB Analysis reveals theoretical properties of ADC and T2 estimation using PBD. The predicted precision and its limiting factors are shown for a representative scenario (SNR˜10) with an RF phase increment of 2°. (a) Predicted standard deviation for ADC, calculated with the amplitude‐weighted and equal‐variance noise models, and the variance‐amplification ratio, where red (*r* > 1) indicates amplitude‐limited precision and dark blue (*r*˜1) indicates conditioning‐limited precision. (b) The corresponding precision and variance‐amplification ratio for T2 estimation. The white mark indicates the optimal parameters for ADC estimation, while the red mark indicates the parameters used.

Figure 6 presents the CRLB identifying the factors limiting ADC and T2 precision. The analysis for ADC estimation (Figure 6a) shows that optimal precision is achieved with a flip angle of approximately 20° and a high gradient moment (˜16π) ratio at both $SD_{\mathrm{amp}}$ and $SD_{\mathrm{eq}}$. $SD_{\mathrm{amp}}$ is optimal at a flip angle of 17° and a gradient moment of 15π (white X), yielding a predicted SD of 440 μmm^2^/s. The experimental parameters used in this study (Red X: 20°, 14π) yielded a comparable predicted SD of 456 μmm^2^/s. The corresponding variance‐amplification ratio reveals that precision is predominantly amplitude‐limited (*r* > 1) when using high gradient moments, but becomes conditioning‐limited (*r*˜1) at low gradient moments. For T2 estimation (Figure 6b), the experimental parameters yielded a predicted SD of 21.4 ms, compared to 18.9 ms at the optimal point. Importantly, the variance‐amplification ratio for T2 is uniformly close to one, indicating that precision is consistently conditioning‐limited.

### 4.4 Phantom Experiments

**FIGURE 7:**
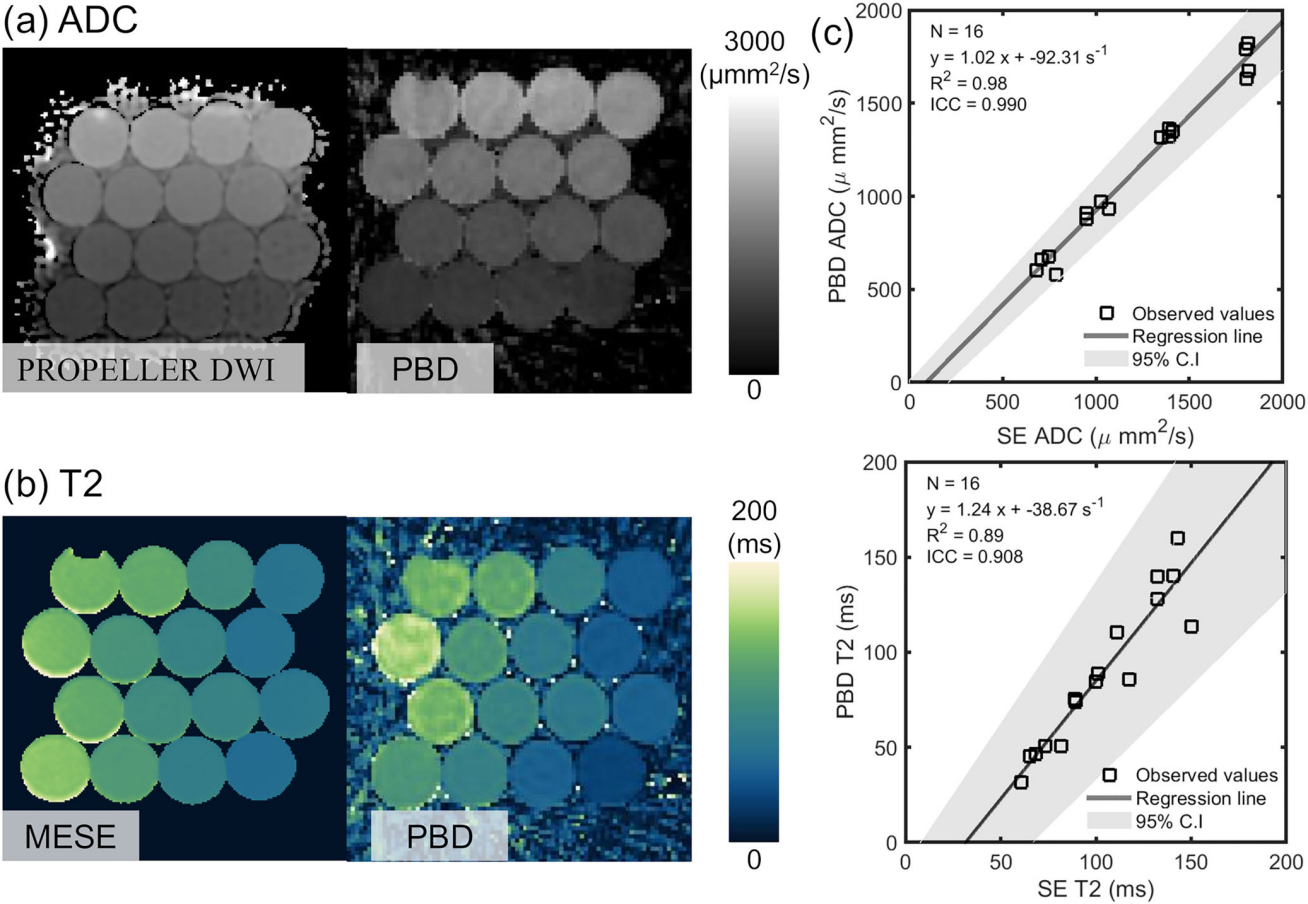
Phantom results indicate excellent agreement of ADC and T2 values between PBD and conventional methods. Example (a) ADC and (b) T2 maps obtained from the PVP phantom using the proposed PBD sequence. (c) Quantitative comparison plots confirm excellent agreement for ADC and strong correlation for T2. A systematic bias is noted for T2, with PBD tending to underestimate lower T2 and overestimate higher T2 relative to the MESE reference.

The PBD method was validated in the phantom vials with varying concentrations of PVP and MnCl_2_ to modulate ADC and T2, respectively. Figure 7 displays example ADC (Figure 7a) and T2 (Figure 7b) maps obtained from the phantom using the PBD. Quantitative accuracy was assessed by comparing PBD measurements against reference methods: PROPELLER diffusion‐weighted imaging (DWI) for ADC and MESE for T2. Scatter plots in Figure 7c show the correlation between PBD estimates and reference values for ADC and T2 across the different vials. Good agreement was observed for ADC (*R* ^2^ = 0.98) values between PBD and the PROPELLER. For T2, despite strong correlation (*R* ^2^ = 0.89), the PBD method showed a tendency to underestimate at low T2 and slightly overestimate at high T2 values compared to the MESE reference in this phantom experiment. PBD underestimated T2 values by an average of 26% over MESE.

**FIGURE 8:**
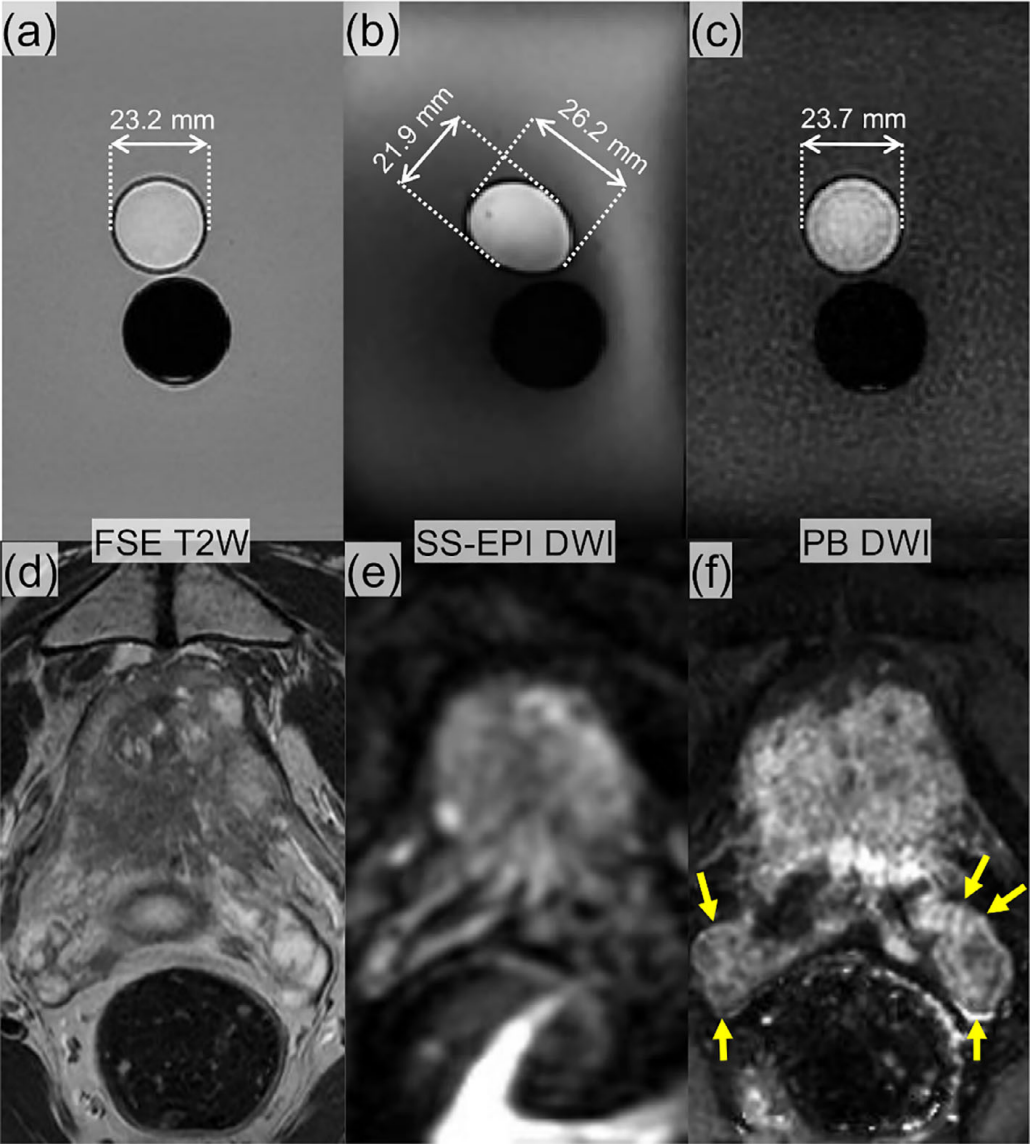
Comparison of geometric fidelity between PBD and SS‐EPI in a phantom and a patient. (a–c) A prostate phantom containing a water/PVP cylinder (top) and an air cylinder (bottom) to mimic the rectum interface. (b) The SS‐EPI (b = 1500 s/mm^2^) shows significant susceptibility‐induced distortion of the top cylinder (21.9 mm × 26.2 mm). (a) PBD preserves the circular geometry (23.7 mm diameter), closely matching (c) the anatomical reference T2W FSE (23.2 mm diameter). (d–f) Representative images from a patient with BPH. (e) The SS‐EPI diffusion weighted image shows severe distortion and blurring at the prostate‐rectum interface and poor visualization of the seminal vesicle (yellow arrows). (d) The PBD weighted image demonstrates superior geometric fidelity with anatomical details that align with (f) the T2W FSE reference.

The prostate phantom experiments demonstrated severe susceptibility‐induced geometric distortions inherent to SS‐EPI and the robustness of PBD to these effects (Figure 8a–c). The air‐filled cylinder created a strong local field inhomogeneity, similar to the gas‐filled rectum. In the SS‐EPI DWI, the prostate‐mimicking cylinder was distorted significantly, measuring 21.9 mm (short axis) by 26.2 mm (long axis). In contrast, the PBD DWI preserved the circular geometry of the cylinder with a diameter of 23.7 mm, which showed excellent agreement with the T2W FSE reference (23.2 mm).

### 4.5 In Vivo Experiments

In the patient with BPH (Figure 8d–f), SS‐EPI showed severe geometric distortion, particularly at the posterior interface, compromising the delineation of the peripheral zone and both seminal vesicles. Conversely, PBD demonstrated high geometric fidelity, producing distortion‐reduced diffusion‐weighted images that maintained anatomical boundaries and mostly aligned with the T2W reference images.

**FIGURE 9:**
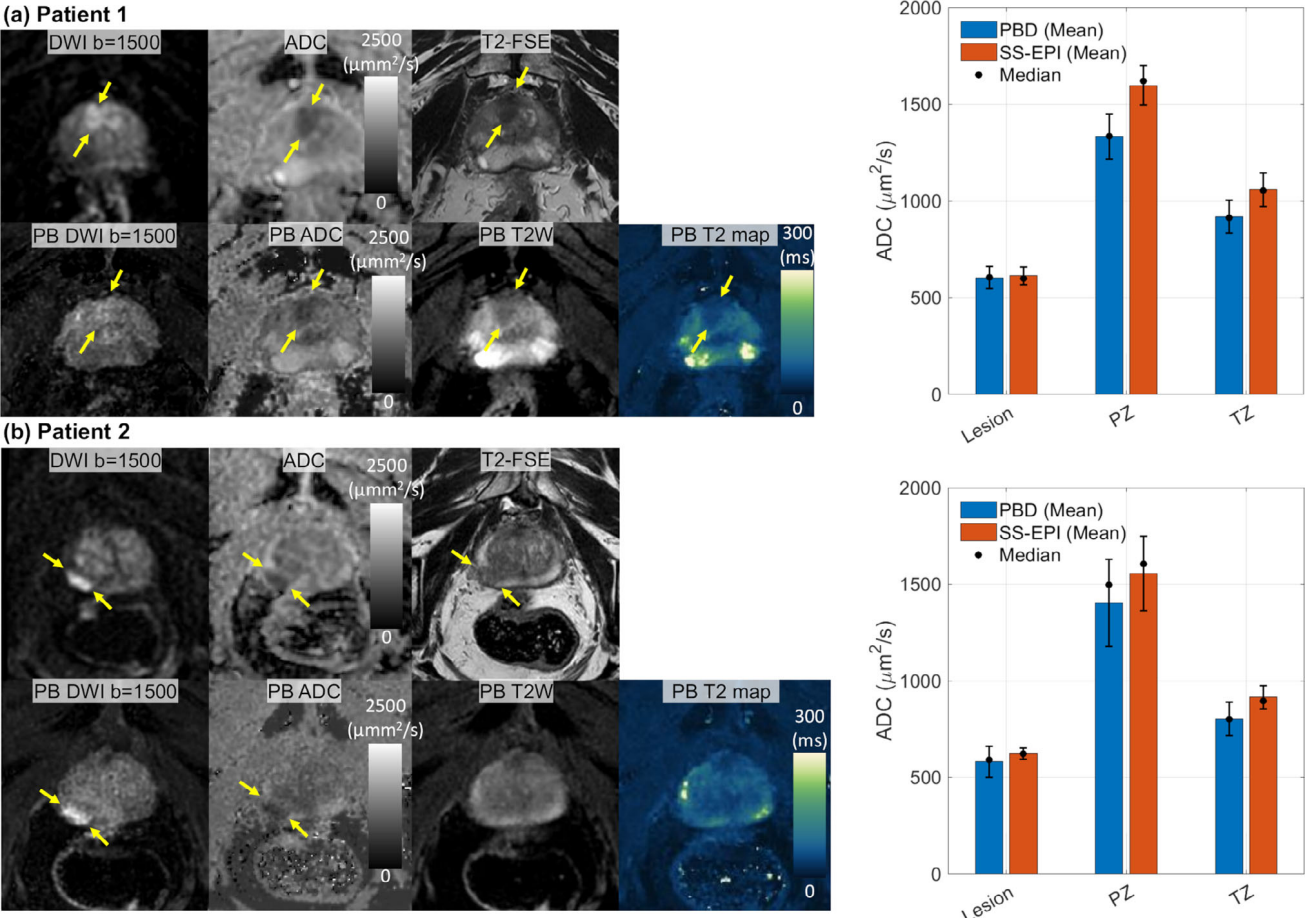
(Left) In both patients (a) 1 and (b) 2, the proposed PBD method provides high‐resolution ADC maps and synthetic diffusion‐weighted images with reduced geometric distortion, successfully identifying suspected cancer foci (arrows). (Right) Quantitative analysis shows that PBD‐derived ADC values in the peripheral zone (PZ) and transition zone (TZ) were underestimated by 10%–20% compared to SS‐EPI. A slight underestimation in suspected lesions resulted in a slightly reduced lesion‐to‐background contrast in these cases.

Figure 9 demonstrates a potential clinical application of the proposed PBD method in two patients with lesions with a high probability of clinically significant prostate cancer (score 5 on Prostate Imaging Reporting and Data System, PI‐RADS v 2.1 [48]). The figure shows ADC maps and corresponding calculated diffusion‐weighted images obtained using PBD. The PBD acquisition provides ADC mapping with relatively high spatial resolution compared to standard clinical SS‐EPI protocols. Suspected foci of cancer, indicated by arrows, were clearly visualized, demonstrating low signal on the ADC map and high signal on the calculated DWI, consistent with restricted diffusion expected in malignant tissue. In both patients, PBD‐derived ADC values in PZ, and TZ were underestimated by approximately 10% compared to SS‐EPI. ADC in suspected lesions showed slight underestimation. Consequently, while PBD provided high‐resolution maps with reduced geometric distortion, this led to slightly reduced lesion‐to‐background contrast compared to SS‐EPI in these two cases. This example highlights the potential of PBD to provide high‐resolution quantitative diffusion information relevant for oncologic imaging.

## 5 Discussion

In this work, we have successfully developed a novel RF‐phase modulated quantitative diffusion and T2 mapping method using 3D radial SoS GRE acquisitions. The technique is based on a new closed‐form solution that we validated against Bloch equation simulations. Furthermore, the method demonstrated excellent quantitative agreement in phantom experiments for both ADC and T2 mapping. The feasibility of this approach was also demonstrated in vivo.

While SS‐EPI remains the gold standard for DWI due to the robustness of bulk motion, PBD offers a distinct advantage in the pelvis where susceptibility‐induced distortion limits EPI. Our results demonstrate that PBD yields ADC maps with high geometric fidelity that align with anatomical T2‐weighted scans, effectively mitigating the distortions observed in standard SS‐EPI. Thus, PBD serves as a high‐resolution alternative for body oncology applications where spatial precision is most important.

Differences between conventional and PBD‐derived quantitative values were observed. Potential contributors to discrepancies with spin‐echo methods may include the effect of T2*, B1+ inhomogeneities, magnetization transfer (MT) effects, residual streaking and motion artifacts, and subtle interactions between the diffusion encoding and T2 estimation within a non‐Gaussian diffusion model. Although the effects of T2* decay should be minimal in the prostate, bias may occur near strong susceptibility gradients (e.g., close to paranasal sinuses). The effects of MT can arise with GRE imaging even without explicit off‐resonance saturation pulses, particularly in myelin‐rich white matter [49], due to repeated RF pulses with a short TR. Our binomial water‐excitation pulses may also accentuate MT weighting, which can alter steady‐state pathway weights and mildly bias estimates if not modeled. We did not model MT, a potential source of bias; extending the model to include MT is a subject for future work. Like other quantitative GRE‐based methods, PBD is sensitive to B1+ field inhomogeneity. Our simulations indicated that uncorrected B1+ inhomogeneity can lead to substantial bias in ADC and T2 estimates. While B1+ correction is recommended in cases of severe B1+ inhomogeneity, its implementation was beyond the scope of this initial feasibility study. Incorporating robust B1+ correction strategies is a key direction for future work. Furthermore, the fundamentally different diffusion encoding mechanisms between PBD and conventional techniques are a likely contributing factor. In conventional SS‐EPI DWI, the diffusion time (Δ), defined as the interval between diffusion‐sensitizing gradient pulses, is a key parameter that affects the sequence's sensitivity to tissue microstructure, including anisotropy. In contrast, the PBD signal (like DW‐DESS) is a composite of multiple configuration pathways, each experiencing a different effective diffusion time. This results in a mix of signals with diffusion weighting from different signal pathways. Further investigation is also needed to fully characterize the accuracy of T2 quantification. Overall, these results indicate a promising new approach for robust 3D GRE‐based mapping of both diffusion and T2.

A key theoretical contribution of our proposed method is the derivation of a novel closed‐form analytical solution (Equation 1) describing the RF‐modulated GRE signal in the presence of diffusion. Analytical solutions for RF‐spoiled GRE sequences without diffusion are well established. Analytical solutions are more computationally efficient and may provide insight into signal behavior compared to numerical simulation‐based methods [25, 50, 51]. In general, incorporating diffusion effects governed by the Bloch‐Torrey [52] equation into analytical models poses challenges, and general closed‐form solutions simultaneously accounting for RF spoiling and diffusion have remained elusive. Our derived solution, validated against Bloch equation simulations, provides an accurate and efficient means to model the complex signal phase behavior in our PBD sequence, enabling parameter estimation using the iterative algorithm.

Our Monte Carlo simulations demonstrated noise properties of PBD and the importance of the choice of statistic for ROI analysis in low‐SNR conditions. The non‐linear estimation in PBD can lead to a skewed, non‐Gaussian distribution of parameter estimates, which substantially biases the mean. In contrast, the median remains a robust and unbiased estimator across all tested SNR levels. This behavior is not unique to PBD and is a known characteristic of other quantitative MRI methods that rely on non‐linear fitting, such as proton density fat fraction (PDFF) estimation [53]. Therefore, the median can be the more reliable metric for PBD in challenging conditions.

The implementation of a SoS trajectory provided sufficient motion robustness for pelvic imaging in our cohort. This finding is consistent with previous studies using center‐out trajectories, such as 3D cones, which have demonstrated reduced motion artifacts in breast DW‐DESS [14]. However, unlike the prior qualitative approach, the proposed PBD method leverages signal phase to derive quantitative ADC and T2 maps.

Further implementation and validation of these methods may provide an important alternative to conventional spin echo EPI‐based diffusion imaging. Conventional SS‐EPI suffers from geometric distortions, limited resolution, and image blurring, which can compromise image quality. These factors compromise image quality and hinder lesion delineation. GRE acquisitions are significantly less prone to the geometric distortions and blurring characteristic of long EPI readouts, potentially enabling higher fidelity and higher resolution imaging. Furthermore, PBD offers joint estimation of ADC and T2. Our noise simulations suggest PBD is robust to Rician noise floor bias, yielding more accurate median ADC estimates, although potentially with lower precision under noise‐dominant conditions. While PBD requires multiple acquisitions and a more complex iterative reconstruction compared to SS‐EPI, its potential for reduced artifacts, improved geometric accuracy, increased spatial resolution, and robust quantification positions it as a promising technique warranting further investigation and clinical evaluation as a complement or alternative to standard SS‐EPI approaches.

While PBD offers superior geometric fidelity and sharpness compared to SS‐EPI, this comes at the cost of SNR. The perceived sharpness of PBD reflects its higher spatial resolution compared to the SS‐EPI protocol used in this study. Furthermore, SS‐EPI images may suffer from inherent T2* decay along the echo train, which can blur spatial detail in the phase encoding direction.

A major difference between conventional DWI and the proposed PBD method is that conventional high *b*‐value images are naturally both diffusion‐ and T2‐weighted, leading to T2 “shine through.” PBD, however, effectively reduces the coupling of diffusion and T2 effects. Because the PBD‐derived T2‐weighted images exhibit weaker T2 contrast, the resulting synthesized high *b*‐value PBD images are predominantly diffusion‐weighted and less susceptible to T2 confounding. This difference in appearance may require accommodation by radiologists, or the introduction of T2 weighting into the high *b*‐value PBD images.

The proposed approach has several limitations. PBD is an unbalanced, coherent steady‐state GRE method and is therefore sensitive to motion, similarly to DW‐DESS. With such sequences, multiple configuration pathways contribute to the resulting signal. Partial RF spoiling attenuates but does not eliminate higher‐order pathways. Accordingly, PBD and DW‐DESS are expected to have similar motion sensitivity; any apparent robustness likely reflects the SoS readout rather than the encoding itself. The four sequential passes could introduce misregistration, a challenge also faced by conventional DWI. Scan time and motion sensitivity may be improved by reducing the number of passes (e.g., to three with two diffusion‐weighted RF‐modulated states) or optimizing sequence parameters. Finally, in fluid‐like tissues (long T2, high ADC) and in the presence of flow, spoiler‐induced intravoxel phase dispersion attenuates the coherent signal, which may bias estimates of ADC and T2.

Partial RF spoiling affects both the phase and the magnitude of GRE signals, and magnitude differences have been used for T2 mapping in a prior work [54]. As shown in the Theory section, the magnitude ratio mainly reflects changes in the real component. Therefore, our phase‐based approach provides the same underlying sensitivity and avoids the low‐SNR Rician floor that typically occurs with conventional magnitude‐based diffusion mapping methods. The signal magnitude still contains useful information, and complex fitting may be a promising extension.

Although many distortion‐correction methods [55] have been proposed for SS‐EPI, such as reversed phase‐encoding [56, 57], field mapping [58], and deep learning‐based approaches [59], they are not routinely used clinically and have practical limitations. Severe susceptibility at air–tissue interfaces (e.g., prostate–rectum) or the presence of metal (e.g., hip prostheses) causes compression/pile‐up and signal loss that cannot be uniquely recovered; corrections add time and complexity [60, 61]. In the pelvis, large field gradients from rectal air or metallic implants degrade co‐registration with T2‐weighted images and reduce diagnostic confidence, motivating distortion‐robust acquisitions such as PBD.

The proposed reconstruction uses weak TV and L2 regularization to stabilize the ill‐conditioned fit in low‐SNR regions and suppress outliers. While such priors reduce noise, they can introduce smoothing and bias; both are nevertheless often used in quantitative MRI (e.g., T1 mapping [62], DTI [63], QSM [64]). We therefore used small weights for TV and L2, and our analysis (Supporting Information S3) shows they provide needed stabilization with minimal impact on ADC and T2 in structured tissue.

Further evaluation will be needed to determine the potential clinical utility of this technique. Finally, we note that this approach assumes free diffusion within the sample Gaussian distribution. This is similar to conventional diffusion MRI that also assumes Gaussian‐based diffusion. The microstructure of many tissues, however, has a variety of anisotropic barriers that will require modification of these models to account for non‐Gaussian effects. We also note that the technique described here should also be amenable to investigating diffusion anisotropy, including diffusion tensor imaging (DTI), such as fiber mapping within the brain.

In conclusion, we developed and demonstrated the feasibility of a novel RF‐phase modulated GRE method for joint ADC and T2 mapping. Although preliminary, the results are promising, and future work is needed to optimize and implement this strategy for clinical care.

## Funding

This work was supported by the National Center for Advancing Translational Sciences (UL1TR002373).

## Conflicts of Interest

Dr. Scott B. Reeder is a founder of Calimetrix LLC, a company focused on developing quantitative MRI phantoms based on technology developed in his laboratory at UW‐Madison. This represents a potential conflicts of interest. Dr. Diego Hernando is a founder and part owner of Calimetrix LLC. Calimetrix is a company that manufactures quantitative MRI phantoms, which represent a potential conflicts of interest due to the overlap between the company's mission and the research interests presented in this paper. The other authors declare no conflicts of interest.

## Supporting information

Data S1. mrm70312‐sup‐0001‐Supinfo.docx.

## Data Availability

The data that support the findings of this study are openly available in phase_based_diffusion_code at https://github.com/dtamadauw/phase_based_diffusion_code.
