# Supplementary material for: Quantitative Diffusion and T2 Mapping Using RF‐Modulated Phase‐Based Gradient Echo Imaging
Source: Magn Reson Med. 2026 Mar 11;96(1):49–65. doi: 10.1002/mrm.70312 (PMC13156447; doi:10.1002/mrm.70312)
Supplement: Supplementary file 1 — Data S1. mrm70312‐sup‐0001‐Supinfo.docx. [file MRM-96-49-s001.docx]

**Supporting Information**

Quantitative Diffusion and T2 Mapping Using

RF-Modulated Phase-Based Gradient Echo Imaging

Daiki Tamada, Ali Pirasteh, David F. Jarrard, Diego Hernando, Scott B. Reeder

# APPENDIX S1

## Analytical solution

In this study, we will derive the steady-state solution for RF-Spoiled Gradient Echo (SPGRE) with diffusion by modifying an approach introduced by Sobol et al(1). The complex magnetization vector $x$ after the *n*th RF pulse is defined as follows.

|  | $\left. \vert x_{n} \right\rangle=\left[ \begin{matrix} F_{n} \\ F_{n}^{*} \\ H_{n} \end{matrix} \right],$ | (1) |
| --- | --- | --- |

where F, and H represent the transverse and longitudinal magnetization, respectively, and $*$ denotes conjugate complex. Assuming a train of RF pulses with transmit phase modulation, the magnetization can be expressed with Fourier expansion as follows.

|  | $F_{n}=\sum_{l=-\infty}^{\infty} f_{l}\left( n \right)e^{j\theta_{l}\left( n \right)}e^{jl\varphi},$ | (2) |
| --- | --- | --- |
|  | $H_{n}=\sum_{l=-\infty}^{\infty} h_{l}\left( n \right)e^{j\vartheta_{l}\left( n \right)}e^{jl\varphi},$ | (3) |

where $f_{l}$ and $h_{l}$ are the complex coefficients in the Fourier expansion of the transverse and longitudinal components of $l$-th configuration, respectively, $\theta_{l}$ and $\vartheta_{l}$ are the phase factors of the $l$-th configuration, and $\varphi$is the phase evolution during TR due to off-resonance effects. Because the longitudinal magnetization is real valued, the below condition must be satisfied:

|  | $\begin{matrix} \vartheta_{-l}\left( n \right) & = & -\vartheta_{l}\left( n \right) \\ h_{-l}(n) & = & h_{l}^{*}(n) \end{matrix}.$ | (4) |
| --- | --- | --- |

When an RF pulse with flip angle $\alpha$ and transmit phase $\phi_{T}$ is used, the relationship between the magnetization before and after excitation can be expressed as

|  | $\left. \vert x_{n} \right\rangle^{+}=\left[ \begin{matrix} {cos}^{2}\frac{\alpha}{2} & sin\frac{\alpha}{2}{\cdot e}^{2j\phi_{T}(n)} & -t\cdot sin^{2}\alpha\cdot e^{j\phi_{T}}(n) \\ {sin}^{2}\frac{\alpha}{2}{\cdot e}^{-2j\phi_{T}(n)} & {cos}^{2}\frac{\alpha}{2} & j\cdot sin\alpha{\cdot e}^{-j\phi_{T}(n)} \\ -\frac{j}{2}sin\alpha{\cdot e}^{-j\phi_{T}(n)} & \frac{j}{2}sin\alpha{\cdot e}^{j\phi_{T(n)}} & cos\alpha\end{matrix} \right]\left. \vert x_{n} \right\rangle^{-},$ | (5) |
| --- | --- | --- |

where the superscripts “-” and “+” denote magnetization before and after excitation. The relationship of configurations is obtained as below using Eq. 2, 3 and 5.

| $\left[ \begin{matrix} f_{l}(n) \\ f_{-l}^{*}(n) \\ h_{l}(n) \end{matrix} \right]^{+}=\left[ \begin{matrix} {cos}^{2}\frac{\alpha}{2} & sin\frac{\alpha}{2}{\cdot e}^{j(\psi_{13}-\psi_{23})} & -t\cdot sin^{2}\alpha\cdot e^{j\psi_{13}} \\ {sin}^{2}\frac{\alpha}{2}{\cdot e}^{-j(\psi_{13}-\psi_{23})} & {cos}^{2}\frac{\alpha}{2} & j\cdot sin\alpha{\cdot e}^{j\psi_{23}} \\ -\frac{j}{2}sin\alpha{\cdot e}^{-j\phi_{T}(n)} & \frac{j}{2}sin\alpha{\cdot e}^{j\phi_{T(n)}} & cos\alpha\end{matrix} \right]\left[ \begin{matrix} f_{l}(n) \\ f_{-l}^{*}(n) \\ h_{l}(n) \end{matrix} \right]^{-},$ | (6) |
| --- | --- |

where

|  | $\begin{matrix} \psi_{13}\left( n,l \right) & = & \phi_{T}\left( n \right)+\vartheta_{l}\left( n \right)-\theta_{l}\left( n \right) \\ \psi_{23}\left( n,l \right) & = & -\phi_{T}\left( n \right)+\vartheta_{l}\left( n \right)+\theta_{-l}\left( n \right) \end{matrix}$ | (7) |
| --- | --- | --- |

To model relaxation effects after the excitation pulse, transverse $X$ and longitudinal $Y$ relaxation operators are introduced as follows

|  | $\left. \vert x_{n+1} \right\rangle^{-}$*=* $\left[ \begin{matrix} X(l)e^{j\phi} & 0 & 0 \\ 0 & X(l)e^{-j\phi} & 0 \\ 0 & 0 & Y(l) \end{matrix} \right]\left. \vert x_{n} \right\rangle^{+}+\left( 1-Y(l) \right)M_{0}\delta_{l,0}\left[ \begin{matrix} 0 \\ 0 \\ 1 \end{matrix} \right],$ | (8) |
| --- | --- | --- |

where $\delta_{l,0}$ is Kronecker’s delta. The relaxation operators, consists of T1, T2, and diffusion relaxation terms, is described by

|  | $\begin{matrix} X(l) & = & {e^{-\frac{t}{T2}}\cdot e}^{-D\gamma^{2}G^{2}t^{3}(l^{2}+l+\frac{1}{3})} \\ Y(l) & = & {e^{-\frac{t}{T1}}\cdot e}^{-D\gamma^{2}G^{2}t^{3}l^{2}} \end{matrix}$ | (9) |
| --- | --- | --- |

where T1 and T2 are spin-lattice and spin-spin relaxation time, and D denotes diffusion coefficient, $\gamma$ is the gyromagnetic ratio, G is the applied gradient field, t is the duration of the gradient field.

Next, we can describe the relaxation effects and phase evolution of each configuration during TR. These effects before and after the RF pulse are given by

|  | $F^{-}\left( n+1 \right)=X(l)e^{i\varphi}F^{+}\left( n \right).$ | (10) |
| --- | --- | --- |

Then, by substituting into Equation 2, we can obtain the Fourier expansion of $F^{-}$ after experiencing TR as:

|  | $F^{-}\left( n+1 \right)=\sum_{l=-\infty}^{\infty} {X\left( l \right)f}_{l}^{+}\left( n \right)e^{j\theta_{l}\left( n \right)}e^{j(l+1)\varphi}$ | (11) |
| --- | --- | --- |

After experiencing relaxation and phase evolution, the $l$-th component of $F^{+}$ will advance from $l$ to $l+1$. This means it becomes the $l+1$-th component in the Fourier expansion of $F^{-}\left( n+1 \right)$. Therefore, $l$-th component of $F^{-}\left( n+1 \right)$ can be expressed as:

|  | $f_{l}^{-}(n+1)={X\left( l-1 \right)f}_{l-1}^{+}\left( n \right)e^{j\theta_{l-1}\left( n \right)}$ | (12) |
| --- | --- | --- |

We can derive the evolution of longitudinal magnetization during TR similarly as:

|  | $h_{l}^{-}\left( n+1 \right)={Y\left( l \right)h}_{l}^{+}\left( n \right)e^{j\vartheta_{l}\left( n \right)}+\left( 1-Y(0) \right)M_{0}\delta_{l,0}.$ | (13) |
| --- | --- | --- |

From Equations 12 and 13, we can obtain the below relationship which explains how the configurations evolve after experiencing TR.

|  | $\left[ \begin{matrix} f_{l}\left( n \right) \\ f_{-l}^{*}\left( n \right) \\ h_{l}\left( n \right) \end{matrix} \right]^{-}$*=* $\left[ \begin{matrix} X(l-1)e^{j(\theta_{l-1}\left( n-1 \right)-\theta_{l}(n))} & 0 & 0 \\ 0 & X(-l-1)e^{j(\theta_{l-1}\left( n-1 \right)-\theta_{l}(n))} & 0 \\ 0 & 0 & Y\left( l \right)e^{j(\vartheta_{l}\left( n-1 \right)-\vartheta_{l}(n))} \end{matrix} \right]\left[ \begin{matrix} f_{l-1}\left( n-1 \right) \\ f_{-l-1}^{*}\left( n-1 \right) \\ h_{l}\left( n-1 \right) \end{matrix} \right]^{+}$  $+\left( 1-Y(0) \right)M_{0}\delta_{l,0}\left[ \begin{matrix} 0 \\ 0 \\ 1 \end{matrix} \right],$ | (14) |
| --- | --- | --- |

In the steady-state situation, the relaxation operator should not depend on $n$. To satisfy this condition, the below relation is obtained.

| $\begin{matrix} \theta_{l}(n) & = & \hat{\theta}\left( n-l \right)+n\tilde{\theta} \\ \vartheta_{l}(n) & = & \hat{\vartheta}\left( l \right)+n\tilde{\vartheta}(l) \\ \phi_{T}(n) & = & A+Bn+Cn^{2} \end{matrix}$ | (15) |
| --- | --- |

Therefore, the evolution matrix to explain consecutive RF pulses can be expressed using Eq. 6, 12, and 13 by

| $\left[ \begin{matrix} f_{l}\left( n \right) \\ f_{-l}^{*}\left( n \right) \\ h_{l}\left( n \right) \end{matrix} \right]^{+}= \left[ \begin{matrix} X(l-1){cos}^{2}\frac{\alpha}{2} & X(-l-1)sin\frac{\alpha}{2}{\cdot e}^{2jCl^{2}} & -t\cdot Y\left( l \right)\cdot sin^{2}\alpha{\cdot e}^{jCl^{2}+2jCl} \\ {X(l-1)sin}^{2}\frac{\alpha}{2}{\cdot e}^{-2jCl^{2}} & {X(-l-1)cos}^{2}\frac{\alpha}{2} & j\cdot Y\left( l \right)\cdot sin\alpha{\cdot e}^{jCl^{2}+2jCl} \\ -X(l-1)\frac{j}{2}sin\alpha{\cdot e}^{jCl^{2}} & X(-l-1)\frac{j}{2}sin\alpha{\cdot e}^{-jCl^{2}} & Y\left( l \right)cos\alpha{\cdot e}^{2jCl} \end{matrix} \right]\left[ \begin{matrix} f_{l-1}\left( n-1 \right) \\ f_{-l-1}^{*}\left( n-1 \right) \\ h_{l}\left( n-1 \right) \end{matrix} \right]^{+}$  $+\left( 1-Y(0) \right)M_{0}\delta_{l,0}\left[ \begin{matrix} 0 \\ 0 \\ 1 \end{matrix} \right],$ | (16) |
| --- | --- |

The relationship of transverse magnetization between two consecutive configurations is obtained by solving linear equations in Eq. 14. In case of $l=0$, the below equation can be derived as follows:

| $f_{0}=\frac{1}{1-Y(0)cos\alpha}\left[ X\left( -1 \right)\cdot\left( 1-Y(0)cos\alpha\right)\frac{f_{-1}+f_{-1}^{*}}{2}+X\left( -1 \right)\cdot\left( cos\alpha-Y(0) \right)\frac{f_{-1}-f_{-1}^{*}}{2}-j\left( 1-Y(0) \right)M_{0}sin\alpha\text{ } \right]$ | (17) |
| --- | --- |

In the case of $l\neq0$, the below matrix form representation is derived.

| $\left[ \begin{matrix} f_{l} \\ f_{-(l+1)}^{*} \end{matrix} \right]=\Omega_{l}\left[ \begin{matrix} f_{l-1} \\ f_{-l}^{*} \end{matrix} \right],$ | (18) |
| --- | --- |

where

| $\Omega_{l}=\frac{1}{X\left( -l-1 \right)\cdot\left( -1+{Y\left( l \right)e}^{2jCl} \right)}\cdot$  $\left[ \begin{matrix} X\left( l-1 \right)\cdot X\left( -l-1 \right)\cdot\left( Y(l)e^{2jCl}-\cos\alpha\right)\cdot{sec}^{2} \frac{\alpha}{2} & -X\left( -l-1 \right)\cdot\left( 1+{Y(l)e}^{2jCl} \right){\cdot e}^{-2jCl^{2}}\cdot{tan}^{2} \frac{\alpha}{2} \\ X\left( l-1 \right)\cdot\left( 1+{Y(l)e}^{2jCl} \right)\cdot e^{2jCl^{2}}\cdot{tan}^{2} \frac{\alpha}{2} & \left( -1+{Y(l)e}^{2jCl}\cos\alpha\right)\cdot{sec}^{2} \frac{\alpha}{2} \end{matrix} \right]$ | (19) |
| --- | --- |

This equation means arbitrary $l$-th configuration can be derived using the matrix recursively as follows.

| $\left[ \begin{matrix} f_{l} \\ f_{-(l+1)}^{*} \end{matrix} \right]={\Omega_{l}\ldots\Omega_{3}\Omega}_{2}\Omega_{1}\left[ \begin{matrix} f_{-1} \\ f_{0}^{*} \end{matrix} \right]$ | (20) |
| --- | --- |

Considering $l\to\infty$, configuration of $f_{l\to\infty}$ should converge to zero because of diffusion relaxation. Therefore, the below equation is obtained.

| $\lim_{l\to\infty} {\Omega_{l}\ldots\Omega_{3}\Omega}_{2}\Omega_{1}\left[ \begin{matrix} f_{-1} \\ f_{0}^{*} \end{matrix} \right]=\Omega_{\infty}\left[ \begin{matrix} f_{-1} \\ f_{0}^{*} \end{matrix} \right]=\left. \vert0 \right\rangle,$ | (21) |
| --- | --- |

where $\Omega_{\infty}$ is the matrix, which is calculated recursively, defined as

| $\Omega_{\infty}=\left[ \begin{matrix} \Omega_{11} & \Omega_{12} \\ \Omega_{21} & \Omega_{22} \end{matrix} \right].$ | (22) |
| --- | --- |

From Eq. 21, the below condition must be satisfied.

| $f_{0}=-\frac{\Omega_{12}}{\Omega_{11}}f_{-1}^{*}$ | (23) |
| --- | --- |
| $\boldsymbol{f}_{\boldsymbol{0}}\boldsymbol{=-}\frac{\boldsymbol{\Omega}_{\boldsymbol{22}}}{\boldsymbol{\Omega}_{\boldsymbol{21}}}\boldsymbol{f}_{\boldsymbol{-1}}^{\boldsymbol{*}}$ | (24) |

Finally, transverse the 0^th^ and -1^st^ configurations, which correspond to FID- and SSFP-echoes, are derived as follows by inserting Eq. 22 into Eq. 17,

| $f_{0}=\beta\left( \eta X\left( -1 \right)+j\left( \eta^{2}-\epsilon\left( X\left( -1 \right)-\epsilon\right) \right) \right),$ | (25) |
| --- | --- |
| $\boldsymbol{f}_{\boldsymbol{-1}}\boldsymbol{=\beta}\left( \boldsymbol{\eta+j}\left( \boldsymbol{X}\left( \boldsymbol{-1} \right)\boldsymbol{-\epsilon} \right) \right)\boldsymbol{.}$ | (26) |

where

| $\epsilon+j\eta=-\frac{\Omega_{22}}{\Omega_{21}},$ | (27) |
| --- | --- |
| $\boldsymbol{\beta=}\frac{\left( \boldsymbol{1-Y(0)} \right)\boldsymbol{M}_{\boldsymbol{0}}\boldsymbol{sin\alpha}}{\left( \boldsymbol{X(-1)-\epsilon} \right)\left[ \boldsymbol{X(-1)}\left( \boldsymbol{cos\alpha-Y(0)} \right)\boldsymbol{+\epsilon}\left( \boldsymbol{1-Y(0)cos\alpha} \right) \right]\boldsymbol{-}\boldsymbol{\eta}^{\boldsymbol{2}}\boldsymbol{(1-Y(0)cos\alpha)}}\boldsymbol{.}$ | (28) |

# APPENDIX S2: Bloch Equation Simulations

Bloch equation simulations were performed to validate the closed form solution of the complex GRE signal with RF phase modulation. We implemented the random walk theory approach proposed by Yarnykh(36) to model diffusion effects in Bloch equation, which accurately models the molecular diffusion weighting induced by gradient pulses in the presence of repeated RF excitation. To evaluate our model, we simulated signals over a wide range of diffusion coefficients (800-3000μm²/s). The simulations were conducted with fixed tissue parameters that represent typical in vivo conditions: T1 of 1000ms, and T2 of 100ms. Also, we assumed TR of 10ms and flip angle of 20° for the acquisitions. To investigate the sensitivity of our method to RF phase modulation, we evaluated four different RF phase increment values (0.5, 1, 2, and 4°). For diffusion encoding, we used two gradient moment values: 2π and 14π (corresponding to low and high diffusion weighting, respectively) with a spatial resolution of 1.0mm. All simulations were performed using identical parameters for both the proposed closed-form equations and Yarnykh's method.


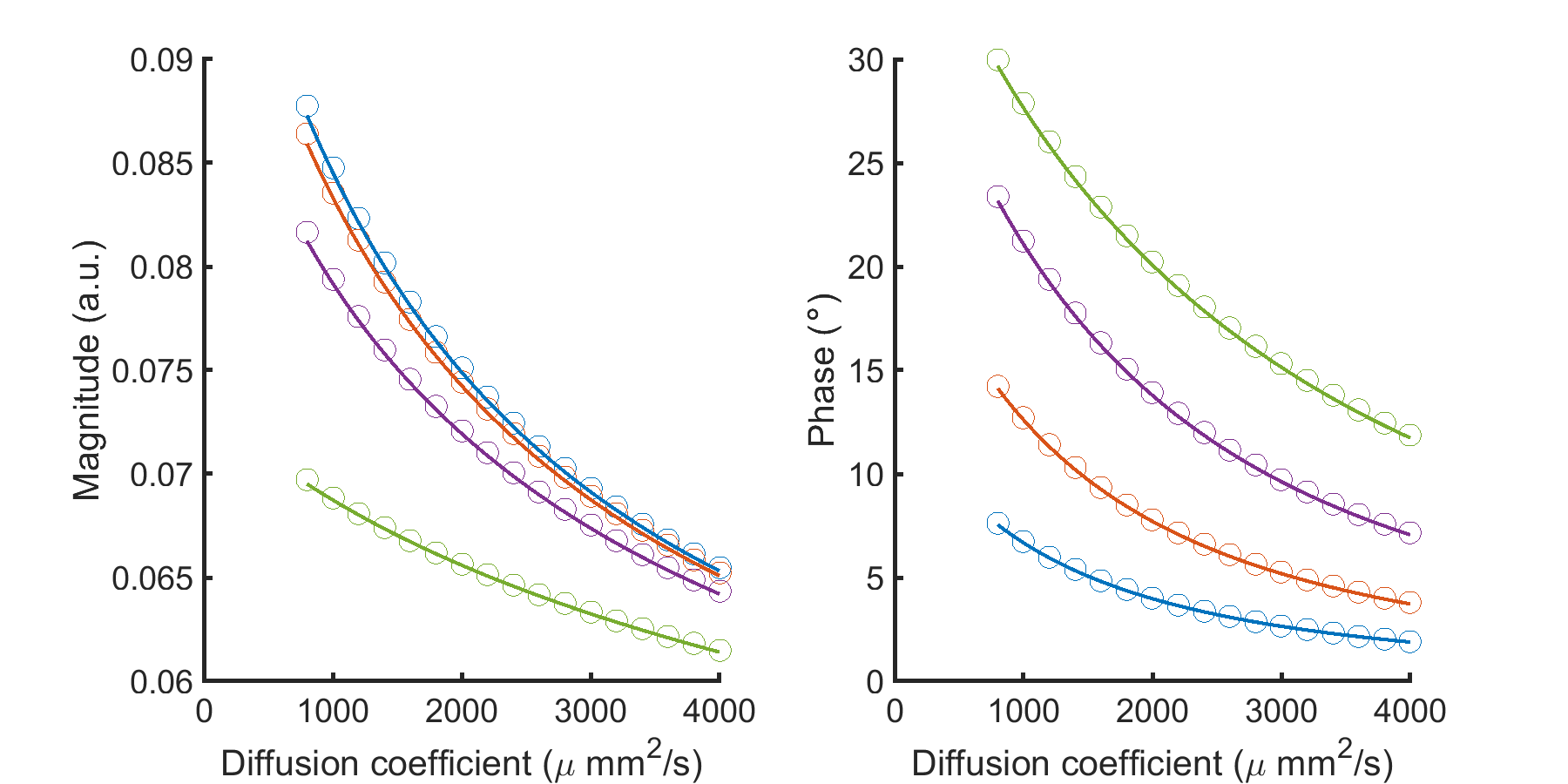


**Figure S2:**Validation of the proposed closed-form analytical solution (solid lines) against Bloch equation simulations (circle markers) incorporating diffusion. Plots show the calculated signal magnitude as a function of diffusion coefficient (800-4000 μm²/s) for different RF phase increments (0.5°, 1°, 2°, 4°), assuming T1=1000ms, T2=100ms, TR=10ms, FA=20°, and high gradient moment (14π). Excellent agreement is observed between the analytical model and the Bloch equation simulations.

# APPENDIX S3: Regularization Coefficients

To validate the choice of regularization weights (λ for TV, β for L2) used for the in vivo reconstructions, we performed a numerical simulation. A 2D phantom with three tissue compartments was created, and noisy PBD signals (SNR=5) were generated. ADC and T2 maps were then reconstructed while varying the λ and β weights.

These results (**Figure S5**) demonstrate the expected trade-off between bias and variance. The small regularization weights used in this study (λ, β $\leq$ 0.5×10^-4^) provide stable estimation without introducing significant quantitative bias. As the weights increase above this range, variability is reduced, but at the cost of an increase in bias. A detailed implementation of this simulation is available in the provided code repository on GitHub.


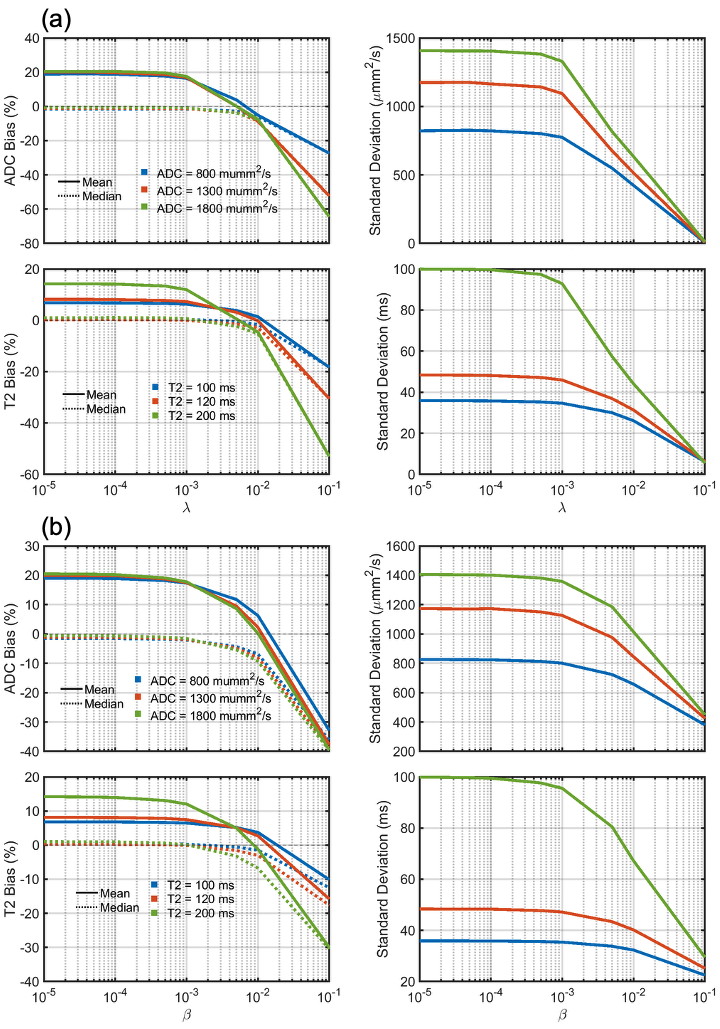


**Figure S5:** Impact of regularization weights on ADC/T2 estimates of PBD. The effect of varying (a) the TV weight (λ) and (b) the L2 weight (β) on the accuracy and precision of ADC and T2 estimates is shown for three simulated tissue types. (Left Column) The relative bias of the mean (solid lines) and median (dotted lines) estimates. For small regularization weights (e.g., $\leq$ 0.5×10^-4^), bias is minimal. As the weights increase, a significant bias is introduced. (Right Column) The standard deviation of the estimates. Standard deviation decreases as the regularization weights increase.

# APPENDIX S4

| Algorithm 1: TV-Regularized T2 and ADC Mapping | | |
| --- | --- | --- |
|  | ***Input:***  ***y_1_, y_2_*** *: phases for high and low gradient moment*  ***λ****: TV regularization parameters*  ***β****: L2 regularization parameters*  ***Γ****: Spline-fitted function*  ***tol****: Convergence tolerance*  ***maxIter****: Maximum iterations* | |
|  | ***Output:***  ***x_1_****: Estimated T2 map*  ***x_2_****: Estimated ADC map* | |
| (1) | ***Initialize Mapz:***  ***x_1_←*** *zeros(rows, cols) // Initial T2 map (0 ms)*  ***x_2_←*** *zeros(rows, cols) // Initial ADC map (0 μmm^2^/s)* | |
| (2) | ***for k = 1 to max_iterations do:*** | |
| (3) |  | *Compute predicted phases and its gradient using lookup tables:*  ***f_1_, f_2_* *←*** *Γ* ***(x_1_, x_2_)*** |
| (4) |  | *Calculate residuals:*  *r_1_* ***←*** *f_1_ - y_1_, r_2_* ***←*** *f_2_ - y_2_* |
| (5) |  | *Compute gradients:*  *grad_data* ***←*** *ComputeDataFidelityGradients(r_1_, r_2_)*  *grad_TV* ***←*** *ComputeTVGradients(x_1_, x_2_)*  *grad_L2* ***←*** *ComputeL2Gradients(x_1_, x_2_)* |
| (6) |  | *Total gradient:*  *grad_x1_* ***←*** *λ·grad_TV _x1_ + β·grad_L2_x1_ + grad_data_x1_*  *grad_x2_* ***←*** *λ·grad_TV_x2_ + β·grad_L2_x2_ + grad_data_x2_* |
| (7) |  | *Update step sizes using Barzilai-Borwein method:*  ***α←****Barzilai_Borwein* |
| (8) |  | *Update maps:*  *x₁* ***←*** *x₁ - α·∇x₁, x₂* ***←*** *x₂ - α·∇x₂* |
| (9) |  | *Check convergence by comparing with the last 5 iterations:*  *if RelativeChange_5iter(x₁, x₂) < tol then*  *break*  *end if* |
| (10) | ***end for*** | |

# APPENDIX S5

To demonstrate the T2 and ADC dependency of the real and imaginary components of the PBD signals, we performed additional simulations. The magnitudes of the real and imaginary signal components were calculated using the closed-form solution (**Equation 1**) across a wide range of T2 (50–300ms) and ADC (0–2000mm^2^/s) values, with variable RF phase increments (0.5°, 1°, 2°, 4°). The magnitude was normalized by the maximum value for each respective phase increment. A fixed TR (10ms) and flip angle (20°) were used, consistent with the parameters in the Theory section.

These simulations demonstrate that magnitude of real component highly depend on T2 and ADC when a large gradient moment is used. Compared to real component, imaginary component has significantly smaller dependency on both T2 and ADC. Those results support the fact that real and imaginary components of the signals are related to echo and FID signals which we demonstrated in previous studies(2,3).


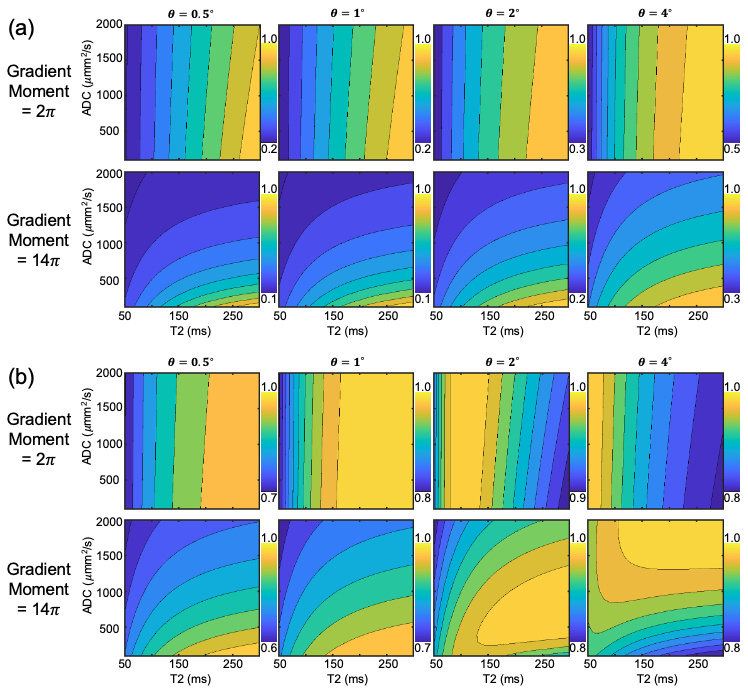
**Figure S5.** Magnitude of the (a) real and (b) imaginary components of the Phase-Based Diffusion (PBD) signal versus T2 and ADC. Simulations were performed for various RF phase increments (θ = 0.5°, 1°, 2°, 4°) and two gradient moments (2π and 14π), using a TR of 10 ms and a flip angle of 20°. Magnitudes are normalized for each phase increment. The real component (a) demonstrates strong T2 and ADC dependence, particularly at the 14π gradient moment, while the imaginary component (b) shows significantly less dependence.

# APPENDIX S6: Effective b-value estimation

**
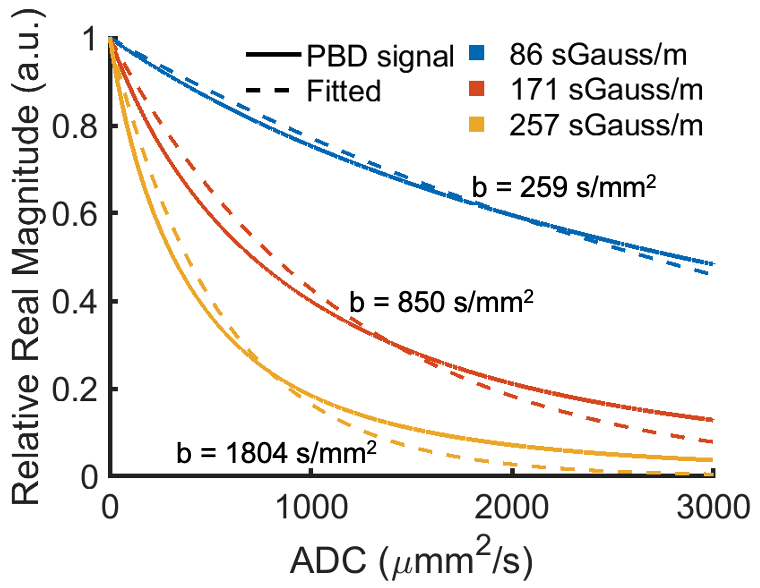
**

**Figure S6**: The b-value for PBD is estimated by fitting the real signal magnitude to the Stejskal–Tanner equation. As shown in the figure, although the signal attenuations do not perfectly fit the equation, this method provides a practical comparison to b-values used in conventional DWI, such as SS-EPI.

# APPENDIX S7:

**Table S11:** Sequence parameters for phantom experiments.

|  | **Phantom** | | |
| --- | --- | --- | --- |
| **Parameters** | **PROPELLER DWI** | **MESE** | **PBD** |
| TR (ms) | 2000 | 1200 | 10.9 |
| (Effective) TE(s) (ms) | 50 | 8.5-68 | 1.5 |
| Flip Angle | 90° | 90° | 20° |
| FOV | 24 × 24 cm^2^ | 18 × 18 × 12.8 cm^3^ | 24 × 24 × 9.6 cm^3^ |
| Matrix Size | 160 × 160 | 256 × 128 | 160 × 160 × 32 |
| Phase-encoding direction | RL | RL | RL |
| Number of Slices | 28 | NA | NA |
| Slice Thickness (mm) | 3 | 4 | 4 |
| Number of Averaging | 1 | 1 | 1 |
| MPG directions | All (3-axis) | NA | RL |
| Bandwidth (Hz/px) | 488 | 488 | 488 |
| b-value (s/mm^2^) | 1000 | NA | NA |
| Gradient Moment  (s·mT/m) | NA | NA | 2.33 /9.33 |
| (Effective)  b-values | 0/1000 | NA | 24/668 |
| Fat Suppression |  |  |  |
| ETL | 1 | 1 | 1 |
| RF phase increment | NA | NA | +2°/-2° |
| Parallel Imaging (PI) |  |  |  |
| Acquisition Time  (min) | 1:28 | 3:25 | 4:25 |

# APPENDIX S8:

**Table S12:** Sequence parameters for in vivo experiments.

| **Parameters** | **SS-EPI**  **DWI** | **FSE T2W** | **PBD** |
| --- | --- | --- | --- |
| TR (ms) | 4500 | 3738 | 10.9 |
| (Effective) TE(s) (ms) | 60 | 106 | 2.9 |
| Flip Angle | 90 | 111 | 20° |
| FOV | 18× 18 cm^2^ | 26× 26 cm^2^ | 18 × 18 × 12.8 cm^3^ |
| Matrix Size | 120×120 | 384×256 | 160 × 160 × 32 |
| Phase-encoding direction | AP | RL | RL |
| Number of Slices | 22 | 43 | NA |
| Slice Thickness (mm) | 4 | 2.4 | 4 |
| Number of Averaging | 5 (b=100)  16 (b=1500) | 1 | 1 |
| MPG directions | 3 axes | NA | RL |
| Bandwidth (Hz/px) | 1953 | 244 | 325 |
| b-value (s/mm^2^) | 1500 | NA | NA |
| Gradient Moment  (s·mT/m) | NA | NA | 2.38/16.3 |
| (Effective)  b-values | 0/1500 | NA | 25/675 |
| Fat Suppression | STIR |  | SSRF |
| ETL | 128 |  | 1 |
| RF phase increment | NA | NA | +2°/-2° |
| Parallel Imaging (PI) | ASSET  (factor 2) | ASSET  (factor 2) |  |
| Acquisition Time  (min) | 4:48 | 2:37 | 4:45 |

# APPENDIX S9:


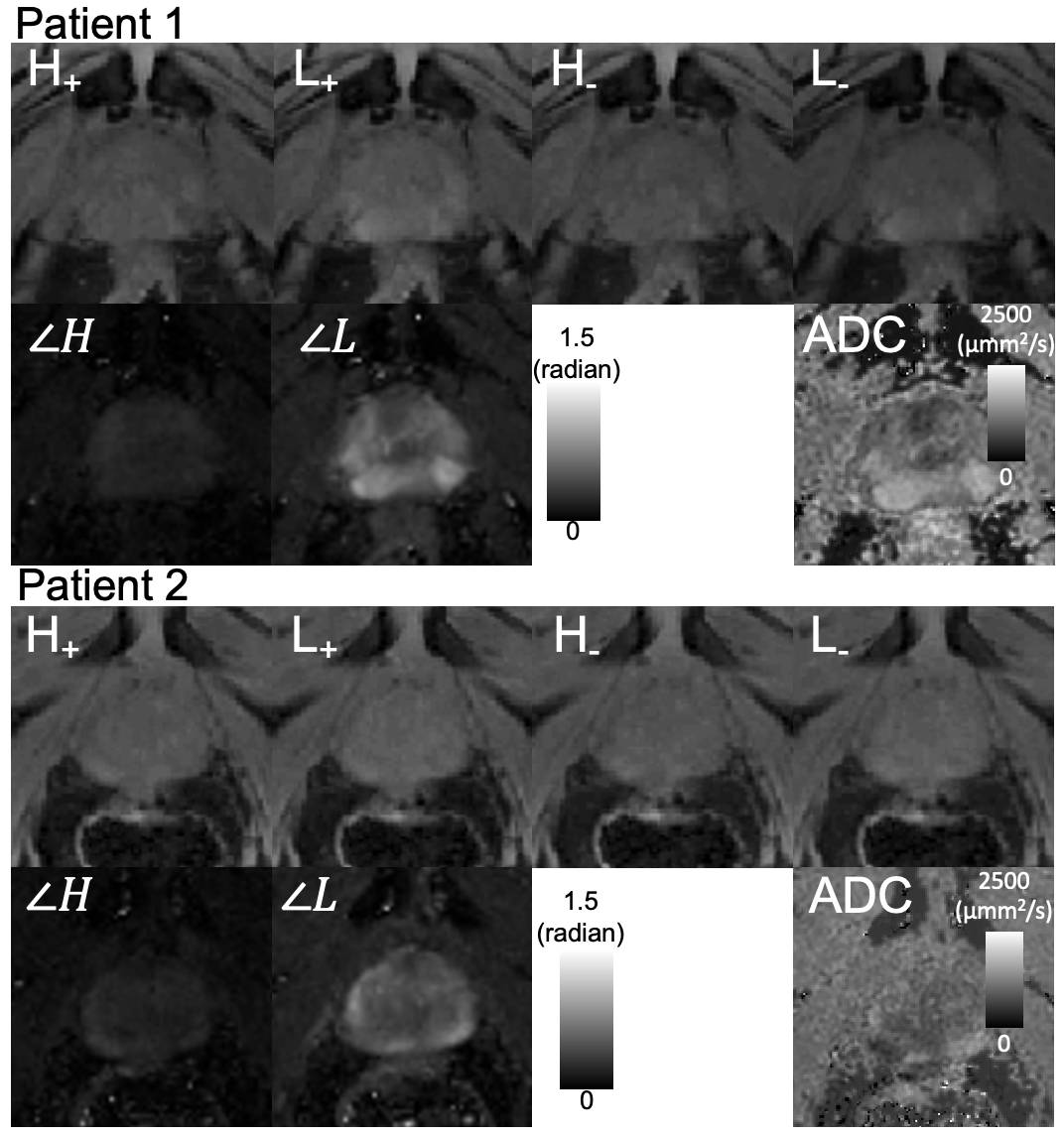


**Figure S14:** Source images and corresponding ADC maps from the PBD acquisition in two prostate cancer patients. For both Patient 1 (top) and Patient 2 (bottom), the figure displays the four individual magnitude images and the final, background-corrected phase maps.

# References

1. Sobol WT, Gauntt DM. On the stationary states in gradient echo imaging. Journal of Magnetic Resonance Imaging 1996;6(2):384-398.

2. Tamada D, Field AS, Reeder SB. Simultaneous T1- and T2-Weighted 3D MRI Using RF Phase-Modulated Gradient Echo Imaging. Magnetic Resonance in Medicine 2021;Accepted on October 25, 2021.

3. Tamada D, Hernando D, Reeder SB. Three-dimensional phase-based diffusion imaging using RF phase-modulated gradient echo imaging with Stack-of-Stars. 2024 May 07, 2024; Singapore. p 2442.

4. Wang H, Cao Y. Spatially regularized T1 estimation from variable flip angles MRI. Medical Physics 2012;39(7Part1):4139-4148.

5. Baust M, Weinmann A, Wieczorek M, Lasser T, Storath M, Navab N. Combined tensor fitting and TV regularization in diffusion tensor imaging based on a Riemannian manifold approach. Ieee T Med Imaging 2016;35(8):1972-1989.

6. Coulon O, Alexander DC, Arridge S. Diffusion tensor magnetic resonance image regularization. Medical image analysis 2004;8(1):47-67.

7. Bilgic B, Chatnuntawech I, Polak D. Chapter 16 - Quantitative Susceptibility-Mapping Reconstruction. In: Akçakaya M, Doneva M, Prieto C, editors. Advances in Magnetic Resonance Technology and Applications. Volume 7: Academic Press; 2022. p 441-467.

8. Calamante F, Gadian DG, Connelly A. Quantification of bolus‐tracking MRI: improved characterization of the tissue residue function using Tikhonov regularization. Magnetic Resonance in Medicine: An Official Journal of the International Society for Magnetic Resonance in Medicine 2003;50(6):1237-1247.
